# RNAi**-**Directed Downregulation of Vacuolar H^+^ **-**ATPase Subunit A Results in Enhanced Stomatal Aperture and Density in Rice

**DOI:** 10.1371/journal.pone.0069046

**Published:** 2013-07-22

**Authors:** Huiying Zhang, Xiangli Niu, Jia Liu, Fangming Xiao, Shuqing Cao, Yongsheng Liu

**Affiliations:** 1 School of Life Science, Chongqing University, Chongqing, China; 2 School of Biotechnology and Food Engineering, Hefei University of Technology, Hefei, China; 3 Department of Plant, Soil, and Entomological Sciences, University of Idaho Moscow, Idaho, United States of America; 4 Ministry of Education Key Laboratory for Bio-resource and Eco-environment, College of Life Science, State Key Laboratory of Hydraulics and Mountain River Engineering, Sichuan University, Chengdu, China; National Taiwan University, Taiwan

## Abstract

Stomatal movement plays a key role in plant development and response to drought and salt stress by regulating gas exchange and water loss. A number of genes have been demonstrated to be involved in the regulation of this process. Using inverse genetics approach, we characterized the function of a rice (*Oryza sativa* L.) vacuolar H^+^-ATPase subunit A (*OsVHA-A*) gene in stomatal conductance regulation and physiological response to salt and osmotic stress. *OsVHA-A* was constitutively expressed in different rice tissues, and the fusion protein of GFP-OsVHA-A was exclusively targeted to tonoplast when transiently expressed in the onion epidermal cells. Heterologous expression of *OsVHA-A* was able to rescue the yeast mutant *vma1Δ* (lacking subunit A activity) phenotype, suggesting that it partially restores the activity of V-ATPase. Meanwhile, RNAi-directed knockdown of *OsVHA-A* led to a reduction of vacuolar H^+^-ATPase activity and an enhancement of plasma membrane H^+^-ATPase activity, thereby increasing the concentrations of extracellular H^+^ and intracellular K^+^ and Na^+^ under stress conditions. Knockdown of *OsVHA-A* also resulted in the upregulation of *PAM3* (*plasma membrane H^+^-ATPase 3*) and downregulation of *CAM1* (*calmodulin 1*), *CAM3 (calmodulin 3*) and *YDA1* (*YODA*, a MAPKK gene). Altered level of the ion concentration and the gene expression by knockdown of *OsVHA-A* probably resulted in expanded aperture of stomatal pores and increased stomatal density. In addition, *OsVHA-A* RNAi plants displayed significant growth inhibition under salt and osmotic stress conditions. Taken together, our results suggest that *OsVHA-A* takes part in regulating stomatal density and opening via interfering with pH value and ionic equilibrium in guard cells and thereby affects the growth of rice plants.

## Introduction

Stomatal pores, surrounded by a pair of guard cells, play a crucial role in controlling gaseous exchange and water release by transpiration [1]. Thus, the development of stomata and the regulation of stomatal apertures are critical for plant survival and productivity. Stomatal aperture is regulated by the reversible swelling and shrinking of guard cells, which sense environmental signals and endogenous hormonal stimuli, such as light, atmospheric CO_2_ levels, humidity, temperature, pathogens and hormones [1], [2]. In response to these stimuli, transport of ions and water through channel proteins across the plasma and vacuolar membranes changes the turgor and volume of guard cell, thereby regulating stomatal aperture [3].

Stomata are produced by a series of cell divisions which starts with an asymmetric division and ends with a symmetric division. The density of produced stomata depends on the frequency of the different kinds of asymmetric divisions [4]. In the initial stage of stomata biogenesis, several genes encoding putative receptors, proteases or kinases, such as *TMM* (*TOO MANY MUTHS*), *SDD1* (*STOMATAL DENSITY AND DISTRIBUTION 1*), *YDA* (*YODA*, a MAPKK gene), have been reported to modulate the frequency and placement of asymmetric divisions [5]. *TMM* encodes a putative cell-surface receptor which is required for stomatal lineage cells to control the number and orientation of the asymmetric of spacing divisions [6]. *SDD1*, encoding Subtilisin protease SDD1 was shown to be expressed in meristemoids and guard mother cells [7]. The loss-of-function mutant *sdd1* exhibited excessive entry divisions but fewer amplification divisions, and subsequently failed to orient spacing divisions [8]. A mutation in *YDA*, encoding a member of *MAPKKKs* which functions upstream of the MKK4/MKK5-MPK3/MPK6 module, resulted in excess production of guard cells by the suppression of asymmetric cell divisions and stoamtal cell fate specification [9]. Downregulation of *OsSIK1*, a putative *RLK* (Receptor-like kinases) gene, was reported to increase stomatal density in adaxial and abaxial leaf epidermis in rice [10]. Transgenic plants with reduced β-type CDK activity showed a decreased stomtatal index due to inhibition of the early meristemoid division and the satellite meristemoid formation [11]. Transcription factors, probably acting in relatively later stages of stomata initiation and development, have been demonstrated to regulate cell proliferation, guard mother cell cytokinesis and guard cell differentiation [5]. For example, it was reported that the transcription factor FLP (FOUR LIPS) interacted with MYB88 and functioned in restriction of divisions of stomatal cell lineage [12]. Loss-of-function in *DST* (*drought and salt tolerance*), coding for a zinc finger transcription factor, resulted in sharp reduction of stomatal density [13].

In addition, several factors including gene-coding proteins or metabolic products were demonstrated to be involved in the regulation of stomatal opening and closing. *SYP121* (*SYR1/PEN1*), a gene coding for a vesicle trafficking protein, was observed to function in facilitating stomatal opening via activation of the K^+^ channel [14]. *PCK1* encoding an isoform of PEPCK (Phosphoenolpyruvate carboxykinase), a key enzyme involved in malate metabolism, was shown to negatively regulate stomatal conductance [15]. *SGR3* (*shoot gravitropism 3*) was reported to encode the SYP22 syntaxin (syntaxin of plants 22) and function in vacuolar fusion and control of stomatal opening [16]. Interestingly, callose, as a component of the cell wall, appears to participate in regulation of stomatal movement, as a strong mechanical stress perceived by the external periclinal guard cell walls was demonstrated to trigger stomatal closure via inducing callose biosynthesis [17].

Three distinct membrane H^+^ pumps capable of generating pH gradients have been identified in plants [18]. The plasma membrane H^+^-ATPase (PM H^+^-ATPase) is a single polypeptide that plays a key role in transport processes across the plasma membrane and functions in nutrient uptake, intracellular pH homeostasis, cell elongation and leaf movements [19]. Vacuolar H^+^-pyrophosphatases (V-PPases) are single-subunit homodimers that also generate proton gradients in endomembrane compartments by using pyrophosphate (PPi) other than ATP [20]. The vacuolar H^+^-ATPase (V-ATPase) complex, known to be required for embryonic development and cell expansion, specifically acidifies the vacuole and other intracellular trafficking compartments [21], [22]. Ionic equilbrium in guard cells mediated by PM H^+^-ATPase has been demonstrated as an essential factor in the regulation of stomatal opening and closing [23]. In the process of stomata opening, PM H^+^-ATPase acts as a key protein to activate H^+^ efflux from cytosol and hyperpolarize the plasma membrane [24]. Overexpression of *PMA4* (*plasma membrane H^+^-ATPase 4*) in tobacco was shown to increase glucose and fructose content and promote stomatal opening [25]. Moreover, *TsVP* (*Thellungiella halophila vacuolar H^+^-PPase*)-overexpressing cotton plants exhibited increased stomatal conductance compared to WT plants under non-salt stressed conditions [26]. Indeed, V-ATPase is a highly conserved, membrane-bound multisubunit enzyme complex containing 14 different subunits and divided into two subcomplexes, with the cytosolic-side V_1_ functioning in ATP hydrolysis composed of eight different subunits (A–H) which are present in stoichiometry of A_3_B_3_C_1_D_1_E_2_F_1_G_2_H_1-2_ and the membrane-integral V_0_ responsible for proton across-membrane transportation constructed by six different subunits (a, c, c’, c”, d and e) which are present in a stoichiometry of a_1_d_1_e_n_c_4-5_c’_1_c”_1_ [27], [28]. This protein complex has been proved to be involved in several cellular processes and physiological responses, such as membrane trafficking, embryonic development, lateral root development, nutrient storage, and environmental stress tolerance [13], [29]-[32]. In addition, several subunits of V-ATPase complex were characterized in regulation of stomatal conductance in various plant species [33], [34]. In Arabidopsis *det3* (*de-etiolated 3*) mutant derived from downregulation of the subunit C of V-ATPase, the ability of stomatal closure was abolished probably due to the disruption of calcium oscillation [33]. Transgenic introduction of subunit c1 of V-ATPase from halophyte grass *spartina alterniflora* into rice plants resulted in a significant increase of salt stress tolerance accompanied by reduced stomata density and early stage closure of the leaf stomata [34]. In Arabidopsis, subunit B of V-ATPase was recently found to bind to F-actin *in vivo* and regulate actin reorganization, suggesting a potential role of the subunit B in the regulation of stomatal movement [35]. Subunit A, the critical component of V-ATPase protein complex, contains an ATP-binding region and may represent a catalytic reaction center [36]. The transcript level of subunit A of V-ATPase has been shown previously to be induced by salt and osmotic stresses in Arabidopsis and barley [37], [38]. However, its function related to stomatal conductance regulation and physiological homeostasis remains largely unknown. In this study, by using inverse genetics approach, we demonstrated that a rice (*Oryza sativa* L.) vacuolar H^+^-ATPase subunit A (*OsVHA-A*) gene plays an important role in the regulation of stomatal movement and determination of stomatal density, which is associated with increase or inhibition of growth of rice plants under nonstress or salt/osmotic stress conditions, respectively.

## Materials and Methods

### Plant Materials and Stress Treatments

*Oryza sativa* L. cv. Nipponbare was used for transformation material. Primary transformants (T_0_) were first planted in the artificial climate incubators under standard conditions (28°C, 16 h light/8 h dark), and transplanted into the experimental field five weeks later. Wild type (WT) and the transgenic progeny plants were grown side by side. To investigate the expression pattern of Os*VHA-A* in different tissues, root, stem, leaf, intenode and flower were collected from wild type plants and immediately frozen in liquid nitrogen then stored at −80°C until further analyses. For stress treatment, WT and transgenic seeds were surface-sterilized and grown on 0.7% agar in petri dishes in a growth chamber (28°C, 16 h light/8 h dark) for one week and transferred into MS liquid medium for 2 weeks. The 3-week-old seedlings of WT and transgenic lines were transferred into new MS liquid medium grown in growth chamber for stress treatments, including 140 mM NaCl for 12 d, 20% PEG6000 for 21 d. In addition, WT and transgenic seeds were surface-sterilized and grown for 7 days on MS medium containing 0, 100, 140 mM NaCl or 0, 150, 200 mM mannitol for seedling salt and drought tolerance analysis.

### Plasmid Construction

DNA manipulations were carried out by using standard procedures (Molecular Cloning). *OsVHA-A* (GenBank accession no. NM_001064815.1) was amplified by RT-PCR (primers: 5′-CTCGAGGATCCTGATGACCTCACAACCGGAT-3′; 5′-AAGCTTGATGTTCAAGTA GATGGTCATCGT-3′) to construct into pSK-int. The *OsVHA-A* was amplified by PCR and re-cloned into different vectors to generate different functional constructs as following: for RNA interference construct (CaMV35S-OsVHA-A-RNAi), *OsVHA-A* was cloned into pHB (driven by 2 × CaMV35S promoter) at the *Bam*H I and *Sac* I restriction sites (PCR primers: 5′-GAGCTCTGATGACCTCACAACCGGAT-3′, and 5′-CTGCAGATGTTCAAGTAGAT GGTCATCGT-3′); for GFP-fusion construct, *OsVHA-A* was into pHB-GFP vector at *Bam*H I and *Mlu* I sites (PCR primers: 5′-ACGCGTATGTCGTACGATCG CGTCAC-3′, and 5′-GGATCCCCTAGCTTCATCTTCTAGGTTGC-3′) to generate 35S-OsVHA-A-GFP construct [39]; for yeast expression construct, *OsVHA-A* was cloned into pYES2 at *Bam*H I and *Sac* I sites (PCR primers: 5′-GGATCCCTCTTCGCTTCTC CTCTC-3′, and 5′-CGAGCTCGGTTTACACGAATGTGATCCTCAAT-3′) to generate pYES2-Os*VHA-A*.

### Subcellular Localization of OsVHA-A

The 35S-OsVHA-A-GFP fusion protein construct as well as 35S-GFP control vector were transferred into onion epidermal cells by using *Agrobacterium tumefaciens* strain EHA105. The GFP signals were monitored under a laser scanning confocal microscopy (OLYMPUS; FV1000-IX81).

### Yeast Complementation

Yeast expression vector pYES2, a gift from Dr. Mor-ris Manolson (University of Toronto) was used as a control, along with pYES2-OsVHA-A to transform into the yeast wild type strain BY4741 (accession number Y00000; EUROSCARF, Frankfurt) and V-ATPase mutant *vma1Δ* (accession number Y03883). Yeast transformants were selected on SD-Ura liquid medium containing 2% glucose at 30^o^C overnight, and then cultured in liquid YPG (pH 5.5, 2% galactose). After incubation, cell densities were calculated by checking the OD_600_ and adjusted to an equal cell number (OD_600_ at 0.2 in each milliliter) to plant at solid medium of YPG (pH 5.5), YPG (pH 7.5) and YPG (pH 7.5, 100 mM CaCl_2_), respectively.

### Rice Transformation

Transgenic plants were generated by *Agrobacterium tumefaciens*-mediated transformation according to the previously described procedure [40]. Transgenic lines were screened for hygromycin (50 mg/L) resistance and confirmed by PCR using *hpt* (accession No. E00777.1)–specific primers (5'-TAGGAGGGCGTGGATATGTC-3' and 5'-TACACAGCC ATCGGTCCAGA-3'). PCR was performed by using Taq DNA Polymerase (Takara, Dalian, China) in MJ Mini™ PCR (BIO-RAD, Hercules, California, USA), following the instruction given by the manufacturer.

### Real-time RT-PCR

For real-time quantitative RT-PCR, total RNA were extracted using Trizol reagent and the first-strand cDNA was synthesized following the protocol provided by the manufacturer (TransGen Biotech). Primers for real-time quantitative RT-PCR were designed for *OsVHA-A* (5′-GGTGTTTCAGTCCCTGCTCTTG-3′, 5′-CCCATAGAACCAGGAGGAAG.

G-3′), *OsVL* (*Vacuolar H^+^-ATPase subunit A-like*, accession No. NM_001052589.1; 5′-CCAAGTATTCCAACTCCCAAGC-3′, 5′-CACAGACTCTTCACGTCCATCCT-3′), *PMA*3 (*Plasma membrane H^+^-ATPase* 3, NM_001073914.1; 5′-ATGAGTCCATTGCCGCTTTAC-3′, 5′-ATACTTGTGCTCTGGGAATACACC-3′), *CAM*1 (*Calmodulin* 1, NM_001056483.1; 5′-GTTTCTCAACCTGATGGCACG-3′, 5′-CTTGTCAAATACACGGAAGGCTC-3′), *CAM*3 (*Calmodulin* 3, NM_001056483.1; 5′-ACGCAAGATGAAGGACACCG-3′, 5′-TGAAGCCGTTCTGGTCTTTGTC-3′), *YDA1* (a MAPKK gene, NM_001060077.1; 5′-GCACCTCCACGCCTCTGTCT-3′, 5′-TCTCTTTCCAAACTGAGGGCTTAG-3′), and the control *ACTIN* (EU155408.1, 5′-ACCTTCAACACCCCTGCTAT-3′, 5′-CACCATCACCAGAGTCCAAC-3′). The real time quantitative PCR was carried out by using SYBR® Premix Ex Taq TM (TaKaRa, Dalian, China). Thermal cycling consisted of a hold at 95°C for 30 seconds followed by 40 cycles of 95°C for 5 seconds and 60°C for 30 seconds. After amplification, samples were kept at 95°C for 15 seconds. Then keep at 60°C for 15 seconds and the temperature was raised gradually by 0.5°C every 5 seconds to 95°C for 15 seconds to perform the melt-curve analysis. Each sample was amplified in triplicate and all PCR reactions were performed on the iCy-cler®PCR system (BIO-RAD, Hercules, California, USA). REST software was used to quantify the mRNA levels of tested genes with *ACTIN* normalization by the 2-Ct method [41]. To confirm the specificity of the PCR reaction, PCR products were electrophoresed on 1% agarose gel to verify accurate amplification product size.

### Plasma Membrane Protein and Tonoplast Membrane Protein Extraction

Microsomal membrane fractions were extracted from mature seedlings roots according to protocol described before with minor change [31], [42]. Tissue was homogenized with 2.0 mL homogenization buffer (1 mL/g fresh weight [FW]) consisting of 330 mM sucrose, 10% (v/v) glycerol, 5 mM Na_2_EDTA, 0.2% (w/v) BSA, 5 mM ascorbate, 0.2% (w/v) casein, 0.6% (w/v) polyvinylpyrrolidone, 5 mM DTT, 1mM PMSF, 3 µg/ml leupeptin, 1µg/ml pepstatin A and 50 mM Hepes-KOH (pH 7.5). The homogenate was filtered through one layer of Miracloth (Calbiochem) and the resulting filtrate was centrifuged at 13,000× g for 10 min at 4°C. The recovered supernatant then was centrifuged at 80,000× g for 50 min at 4°C. The resulting membrane pellet was the crude microsomal membranes protein.

The plasma membrane protein was extracted according to a previous study [43]. The microsomal pellet was gently resuspended in 330 mM sucrose, 3 mM KCl, 0.1 mM EDTA, 1 mM DTT, 1 mM PMSF, 1 µg/ml leupeptin, 1 µg/ml pepstatin A and 5 mM potassium phosphate (pH 7.8). Resuspended microsomal fractions were mixed with 6.2% (w/w) Dextran T-500 and 6.2% (w/w) polyethylene glycol 3350 in 5 mM potassium phosphate (pH 7.8), 330 mM sucrose, 3 mM KCl. After mixing, the upper phase were collected, diluted with resuspension buffer containing 0.33 M sucrose, 10% (w/v) glycerol, 0.1% (w/v) BSA, 0.1 mM EDTA, 2 mM DTT, 1 µg/ml leupetin, 1µg/ml pepstatin A and 20 mM Hepes-KOH (pH 7.5), then centrifuged at 100,000 g for 50 min. The resulting pellets were plasma membrane protein and then resuspended in the above-described resuspended buffer involving 1 mM EDTA. Moreover, the crude microsomal membrane protein was also used to the extraction of tonoplast vesicles [44]. The microsomal membrane pellet was resuspended in a buffer containing 2 mM BTP/Mes, pH 7.0, 250 mM sucrose, 0.2% BSA, 10% glycerol and 1 mM DTT, covered with a 25/38% (w/w) discontinuous sucrose density gradient, and then centrifuged with 100,000 g for 2 h. The tonoplast protein was removed from the interface.

The concentrations of plasma membrane protein and tonoplast membrane protein were determined by Lowry method [45].

### Evaluation of the Purity of Plasma Membrane and Tonoplast Vesicles

To characterize the purity of the plasma membrane vesicles and isolated tonoplast vesicles, H^+^-ATPase substrate hydrolytic activity was assayed by determining the release of Pi from ATP in the presence or absence of nitrate (NO_3_^-^), vanadate (VO_4_^3-^) and azide (NaN_3_), which are the specific inhibitors of V-, P-, and F-type H^+^-ATPase associated to the tonoplast vesicles, plasma membrane and mitochondria, respectively [46], [47].

### V**-**ATPase, V**-**PPase and Plasma Membrane H^+^ **-**ATPase Activity Assay

Ten micrograms of tonoplast membrane protein was used to examine the V-ATPase and PPase activity with 10 μg BSA as a negative control. The V-ATPase activity was assayed in a reaction medium containing 25 mM Tris-Mes (pH 7.0), 4 mM MgSO_4_× 7H_2_O, 50 mM KCl, 1 mM NaN_3_, 0.1 mM Na_2_MoO_4_, 0.1% Brij 35, 500 μM NaVO_4_, and 2 mM Mg-ATP. V-ATPase activity was calculated as the difference measured in the absence or presence of 100 nM Concanamycin A. PPase activity analysis, based on colorimetrical determination of Pi release [31], was performed after an incubation period of 40 min at 28°C with reaction buffer contained 25 mM Tris-Mes (pH 7.5), 2 mM MgSO_4_× 7H_2_O, 0.1 mM Na_2_MoO_4_, 0.1% Brij 58, and 0.2 mM K_4_P_2_O_7_, then terminated by adding 40 mM citric acid. PPase Activity was expressed as the difference between the measurements in the absence or presence of 50 mM KCl.

Ten micrograms of protein was used to determine the activity of plasma membrane H^+^-ATPase [48]. The reaction buffer contained 3 mM ATP, 2.5 mM MgSO_4_, 50 mM KCl, 1 mM NaN_3_, 0.1 mM Na_2_MoO_4_, 50 mM NaNO_3_ and 33 mM Tris-MES (pH 7.5), with or without 200 µM Na_3_VO_4_ and 0.02% Triton X-100. SDS at 0.2% (w/v) concentration was used to terminate precipitation.

### Proton**-**pumping Assay

Proton transport into vacuole was measured by the quenching of ACMA (9-amino-6-chloro -2-methoxyacridine) fluorescence [44], [49]. The proton pump activity of V-ATPase was assayed in a reaction buffer containing 250 mM sorbitol, 50 mM KCl, 3 mM ATP, 50 μM NaVO_4_, 1 mM NaN_3_, 2 μM ACMA and 10 mM Mes-Tris (pH 7.5). MgSO_4_ (3 mM) was used to initiate the reaction. The proton-pumping activity of the isolated plasma membrane vesicles was measured by monitoring the quenching of ACMA [50]. The reaction medium contained 25 mM K_2_SO_4_, 5.2% (w/v) glycerol, 0.15% Brij 58, 0.2 μM ACMA and 50 mM MES-NaOH (pH 6.5). MgATP (1 mM) was used to initiate the reaction. The fluorescence quenching was measured with an excitation wavelength of 410 nm and an emission wavelength of 480 nm by an auto microplate reader (infinite M200, Tecan, Austria).

### pH Measurements

Vacuolar pH of 3-week-old pot-grown plant was determined using the fluorescent cell-permeable dye BCECF AM (Molecular Probes) [31], [51]. Loading of the dye was performed in liquid media containing 1/10 MS medium, 0.5% sucrose, and 10 mM Mes-KOH (pH 5.8) in the presence of 10 μM BCECF AM and 0.02% Pluronic F-127 (Molecular Probes). After 1 h of staining at 22°C in darkness, roots were washed once for 10 min in the media mentioned above. BCECF fluorescence was detected using an OLYMPUS FV1000 confocal laser-scanning microscope. The images were obtained using the LSM Confocal software and a Plan-Neoﬂuar ×25 water immersion objective. The ﬂuorophore was excited at 488 and 458 nm, respectively, and the emission was detected between 530 and 550 nm. All images were exclusively recorded within the root hair zone of fully elongated cells. Ratio images were generated using the ion concentration tool of the Zeiss LSM Confocal software and the images were processed using Adobe Photoshop software. The ratio values were obtained using the program Image J 1.41 (National Institutes of Health). The integrated pixel density was measured and the values of the 488-nm-excited images were divided by the values of the 458-nm-excited images. The ratio was then used to calculate the pH on the basis of a calibration curve.

### Scanning Electron Microscopy (SEM) Analysis of Rice Stomata

Stomatal apertures were measured as described previously [52]. Leaves of 3-week-old plants treated with 20% PEG6000 for 21 days were detached and immediately fixed by 2.5% glutaraldehyde in 0.1 M phosphate buffer (pH 7.2) at 4°C for 24 h, washed twice with 0.1 M phosphate buffer for 10 min, then immersed in 50, 60, 70, 80, 90, and 100% ethanol sequentially for 10 min each. After drying at vacuum dryer, the samples were coated with gold for scanning electron microscopy (JSM-6490LV) analysis.

### Water Loss Rate

Water loss in transgenic rice plants was measured to determine drought tolerance according to the method reported previously with minor modifications [53]. Ten fully expanded leaves from 21-day-old plants cultured in hydroponic growth medium were excised for immediate measurement of fresh weight, then placed on open petri dishes at 25°C and 48% RH (Relative Humidity). The water loss of each leaf was determined by weighing at 30-min intervals for 2.5 h. Leaf water loss rate was calculated as the weight of water loss at each interval divided by the initial leaf weight.

### Na^+^ and K^+^ Contents Under Salt Stress

Salt-treated wild type and transgenic plants were oven dried for at least 24 h at 80°C and weighed. And then, the material was digested in 69% (v/v) HNO_3_ for 12 h for elemental extraction. Concentrations of sodium and potassium were determined in appropriately diluted samples by atomic absorption spectrophotometry in an air-acetylene flame [54].

### Osmolality and Leaf Conductance

The leaves of PEG6000-treated wide type and transgenic lines were floated on deionized water overnight at 4°C. The leaves were then frozen, thawed, and mechanically disrupted. The osmolality of the resulting sap was measured with a vapor pressure osmometer (5500 WESCOR) [55]. The leaf conductance was monitored on ten leaves per plants with a steady-state diffusion porometer as described [56].

### H^+^ Fluxes

Net H^+^ fluxes were measured by using the non-invasive micro-test system (NMT; BIO-IM, Younger USA Amherst, MA, USA) as described previously [57]. WT and transgenic line *OsV-5* root segments with 2–3 cm apices were rinsed with water and immediately incubated in measuring buffer (0.1 mM KCl, 0.1 mM CaCl_2_, 0.1 mM MgCl_2_, 0.5 mM NaCl, 0.2 mM Na_2_SO_4_, pH 6.0) for equilibration for 30 min and then transferred to the measuring chamber containing 10-15 ml fresh measuring buffer to determine H^+^ fluxes at a distance of 5000 μm away from the root apexes, in which a vigorous flux of H^+^ was usually observed. The concentration gradient of H^+^ was measured by moving the ion-selective microelectrode between two positions (30 μm) in a pre-set excursion at about 5 s per point. A representative curve was prepared and the mean ± SE of the H^+^ value was calculated from three repetitive experiments. For H^+^ fluxes assays under PEG6000 treatment, roots were collected from WT and transgenic line *OsV-5* plants that had been exposed to 20% PEG6000 for 1 d.

## Results

### The Expression Pattern and Subcellular Localization of OsVHA**-**A in Rice

A rice EST (accession no. NP8255364) homologous to Arabidopsis V-ATPase subunit A (accession no. NP10423601) was identified in the TIGR database (www.tigr.org). Using reverse transcription PCR, a 1,863 bp cDNA fragment containing a complete open reading frame (ORF) was obtained and designated as *OsVHA-A* (accession no. NM_001064815.1). Sequence analysis revealed that *OsVHA-A* encoded a protein of 620 amino acids with a predicted molecular weight of 65.62 kDa (Figure S1). Amino acid alignment showed that *OsVHA-A* shared 95.40%, 94.77%, 91.92% and 89.70% identity with the orthologs from *Sorghum bicolor* (accession no. XM_002451594.1), *Zea mays* (accession no. AY104754.1), *Triticum aestivum* (accession no. AK332978.1), *Arabidopsis thaliana* (accession no. NM_001036222.2), respectively, and the homologous proteins derived from *Oryza sativa*, *Sorghum bicolor*, *Zea mays* were closely clustered, whereas those from other species formed several evolutionary branches (Figure S2).

**Figure 1:**
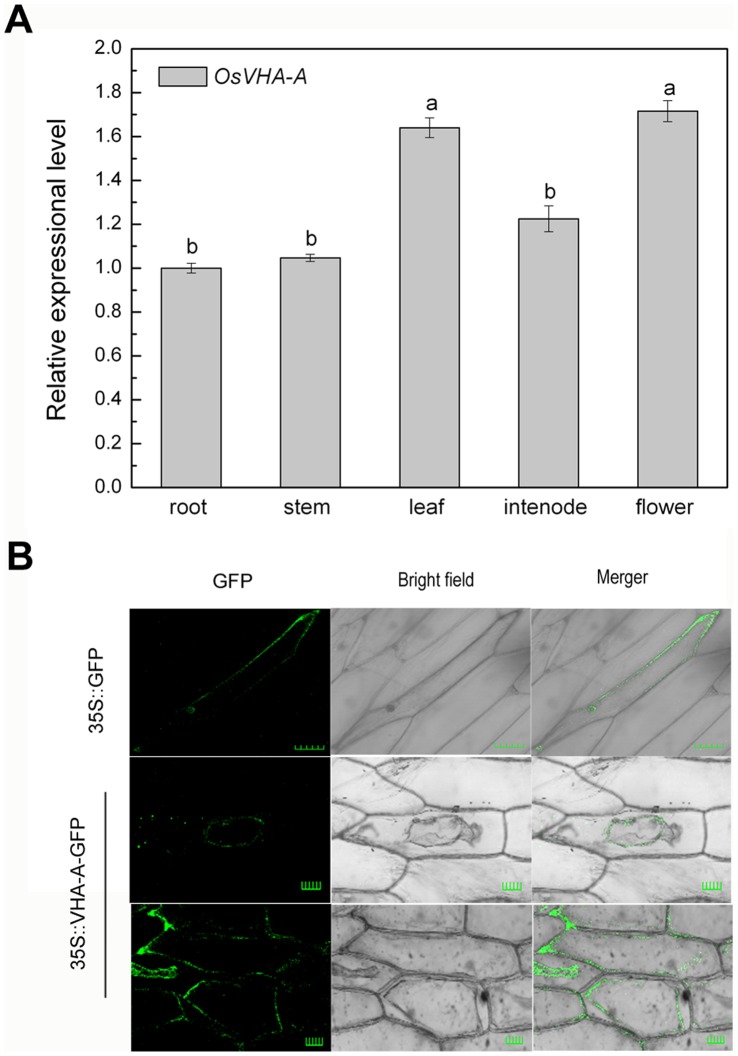
Expression pattern of *OsVHA* *-* *A* in various tissues and subcellular location of OsVHA-A. (A) Expression levels of *OsVHA-A* revealed by real-time quantitative RT-PCR in root, stem, leaf, internode, and flower in rice cv. Nipponbare. *Actin* was amplified as internal control. Different letters indicate statistically significant differences among each tissue (Duncan’s multiple range test, *P*<0.05). (B) Subcellular localization of OsVHA-A-GFP fusion protein in onion epidermal cells with the control of GFP. Transient expression was performed by using *Agrobacterium tumefaciens* (strain EHA105)-mediated infiltration. Scale bars  = 50 µm.

As shown in Figure 1A, differential expression levels of *OsVHA-A* gene were detected in root, stem, leaf, flower and internode from the mature rice plant. The result indicated *OsVHA-A* was expressed constitutively, with greater expression abundance in leaf and flower. According to the subcellular localization of OsVHA-A, green fluorescence signal derived from GFP-OsVHA-A fusion protein was exclusively detected in tonoplast, while green fluorescence signal from GFP alone was visualized throughout the cytoplasm and nucleus (Figure 1B).

**Figure 2:**
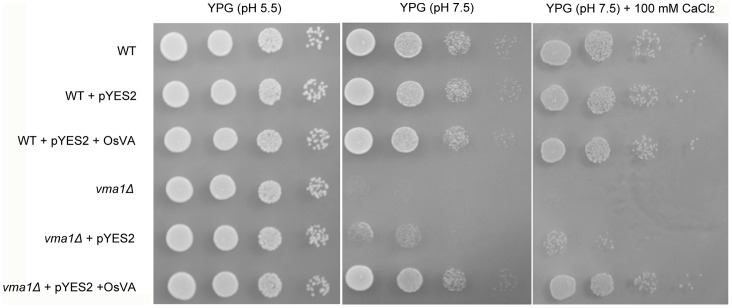
Yeast complementation assay. Wide type (WT) yeast stain BY4741, OsVHA-A ortholog mutant (*vma1Δ*), and yeast transformants with pYES2 vector, pYES2-OsVHA-A (OsVA), respectively, grown on YPG (pH 5.5), YPG (pH 7.5) and YPG (pH 7.5, 100 mM CaCl_2_) solid media. Results shown are representative.

To further test the biochemical activity of *OsVHA-A*, the yeast (*Saccharomyces cerevisiae*) mutant (*vma1Δ*) lacking subunit A of V-ATPase was employed for complementation assay [58]. The mutant *vma1Δ* was shown to grow normally on the YPG medium with physiological pH value buffered to 5.5, but develop poorly when the pH value increased up to 7.5. Furthermore, the mutant *vma1Δ* failed to grow on the medium buffered to pH 7.5 supplemented with 100 mM CaCl_2_, while its cells expressing *OsVHA-A* were capable of restoring the *vma1Δ* phenotype and growing normally (Figure 2). Thus, heterologous expression of *OsVHA-A* appears to partially complement the defective V-ATPase activity in the yeast *vma1Δ* mutant.

### Generation of *OsVHA* *-* *A* *-*RNAi Transgenic Rice Lines

**Figure 3:**
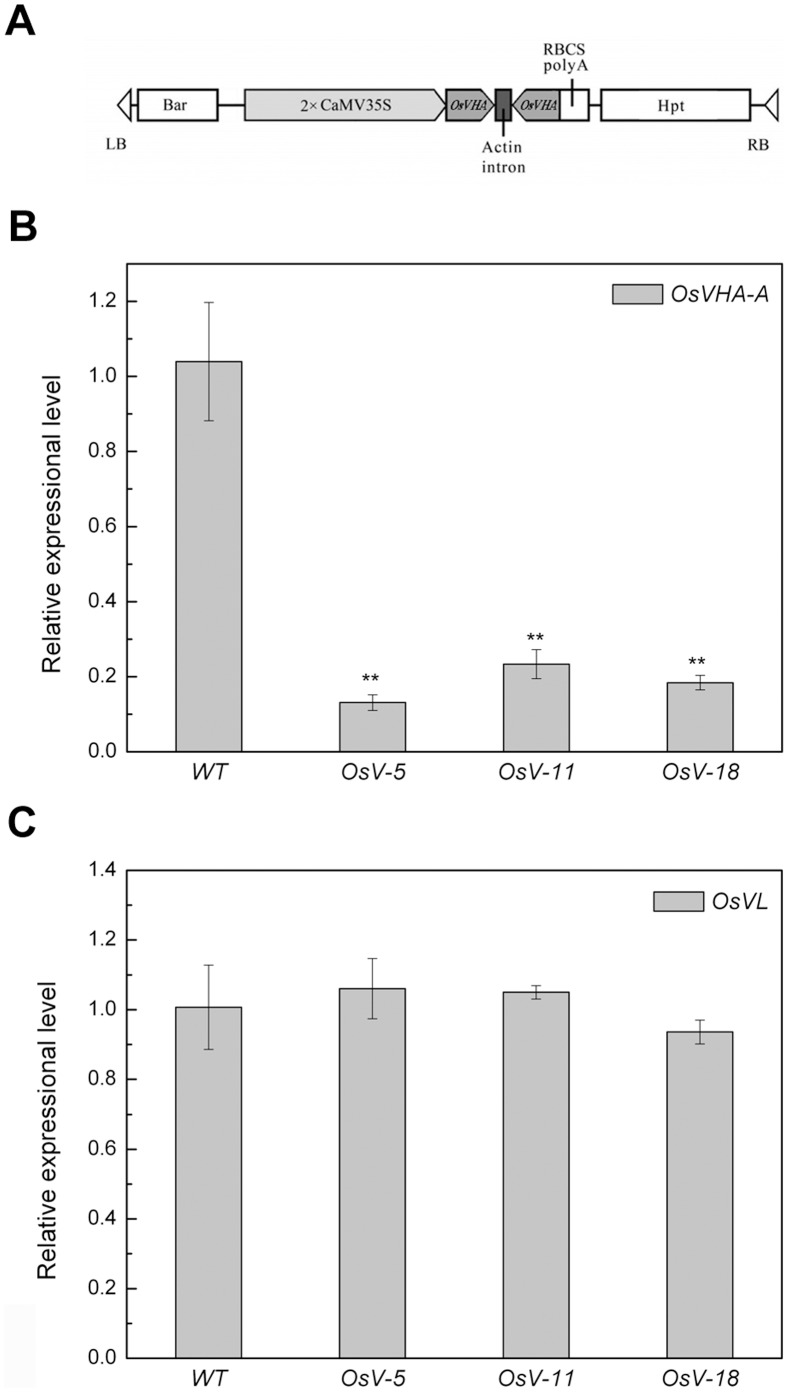
Construct and molecular analysis of WT and transgenic plants. (A) Schematic diagram of part of the T-DNA region of the transforming construct *CaMV-35S-OsVHA-A*-RNAi. Real-time quantitative RT-PCR analysis of *OsVHA-A* (B) and *OsVHA-A*-*like* gene *OsVL* (C) mRNA levels conducted in fully expanded leaves derived from wild-type (WT) and three OsVHA-A RNAi transgenic lines (*OsV-5*, *OsV-11*, and *OsV-18*). Each bar represents three replications from each RNA samples. Asterisks (**) indicate significant differences from WT at *P*<0.01.

To address the functional significance and physiological role of *OsVHA-A*, transgenic rice lines were generated to express *OsVHA-A*-derived inverted repeat sequences under the direction of 35S promoter by using *Agrobacterium*-mediated T-DNA transfer. Primary transgenics (T_0_) resulted in PCR amplification with primers designed to the *hpt* (hygromycin phosphotransferase gene) marker. qRT-PCR analysis showed that the endogenous *OsVHA-A* expression was significantly repressed in the T_2_ homozygous transgenic lines (*OsV*-5, *OsV*-11, *OsV*-18, respectively) (Figure 3 A and B). To verify the specificity of repression in the *OsVHA-A* RNAi downregulation lines, the expression level of *OsVL* (*Vacuolar H^+^-ATPase subunit A-like*, accession No. NM_001052589.1), the homologous gene closest to *OsVHA-A* found in rice genome, was detected, and there was no significant difference between the RNAi lines and WT plants (Figure 3C).

### Knockdown of *OsVHA* ***-*** *A* Leads to Elevated Vacuolar pH Value Through Abrogating Proton**-**pump Activity

**Figure 4:**
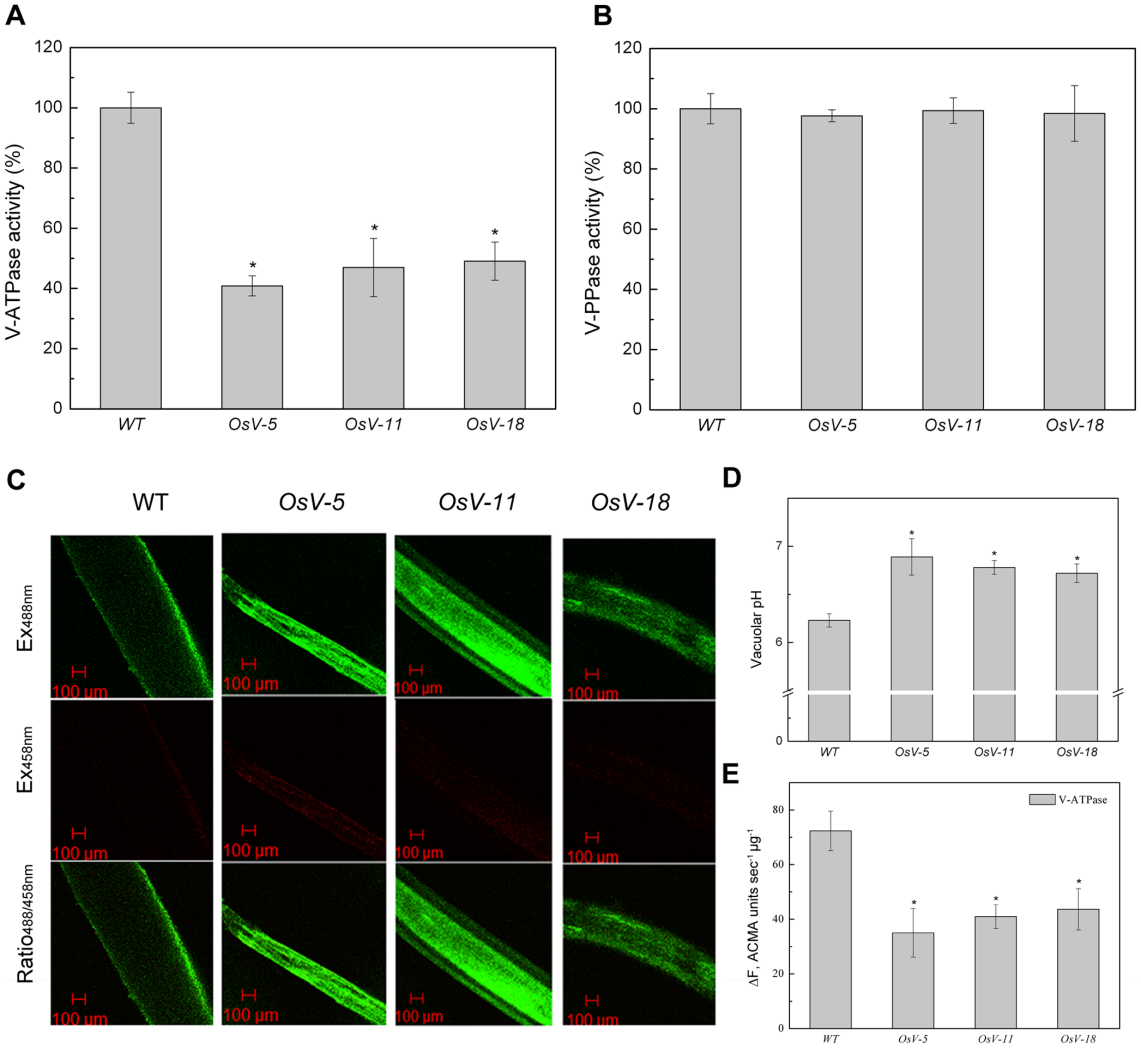
V-ATPase, PPase activity assays and vacuolar pH measurements in *OsVHA* *-* *A* RNAi transgenic lines. (A) Vacuolar H^+^-ATPase activity and (B) V-PPase activity were determined in wild type (WT) and three *OsVHA-A* RNA interference lines (*OsV-5*, *OsV-11*, and *OsV-18*). (C) The images showing emission intensities of vacuoles from epidermal root cells loaded with BCECF AM. Results shown are representative. Scale bars  = 100 μM. (D) The vacuolar pH values calculated from (C). (E) V-ATPase proton-pumping measured by the quenching of ACMA fluorescence. Ten micrograms of tonoplast vesicles were applied to detect fluorescence density. Each bar represents three replications. Asterisks (*) indicate significant differences from WT at *P*<0.05.

To determine whether knockdown of *OsVHA-A* alter the activity of V-ATPase, tonoplasts were isolated from root of 3-week-old rice WT and RNAi transgenic plants. The V-ATPase activity was measured as Concanamycin A-sensitive ATP hydrolysis [31], [59]. As shown in Figure 4A, the V-ATPase activities in all *OsVHA-A* RNAi transgenic plant roots were significantly reduced compared to that in WT. Similar experiments were conducted to examine the activity of V-PPase. The V-PPase activity was measured as KCl-sensitive PPi hydrolysis [31]. No significant difference in the V-PPase activity was detected between WT and *OsVHA-A* RNAi transgenic plants (Figure 4B). These results suggest that knockdown of *OsVHA-A* in the transgenic plants specifically suppressed the V-ATPase activity instead of V-PPase activity.

Next we examined whether the downregulation of V-ATPase activity leads to an increase of vacuolar pH value using confocal laser-scanning microscopy (CLSM) images-based approaches (see Materials and Methods). 2′, 7′-bis-(2-carboxyethyl)-5-(and-6) -carboxyﬂuorescein (BCECF) was employed as a ratiometric pH indicator and its membrane-permeable acetoxymethyl ester was loaded into the vacuoles of intact root tissues from 1-week-old seedlings of WT and RNAi plants. BCECF-stained roots were subjected to analysis by CLSM. Based on the *in situ* calibration curve (Figure S3), the detected emission intensities excited at 488 and 458 nm were converted into pH values (Figure 4C). Vacuoles from the transgenic root cells had a pH fluctuation ranging from 6.7 to 6.9, whereas the pH value in WT root cells was about 6.2 (Figure 4D), indicating that knockdown of *OsVHA-A* inhibited the transport of H^+^ from cytosol into vacuole.

As shown in Figure 4E, the V-ATPase proton-pumping activity in three *OsVHA-A* RNAi transgenic lines were markedly lower than that in WT, indicating that the elevation of vacuolar pH value in *OsVHA-A* RNAi transgenic lines might be attributed to the reduction of V-ATPase proton-pumping activity.

### Knockdown of *OsVHA* ***-*** *A* Promotes the H^+^ Efflux by Increasing Plasma Membrane H^+^ **-**ATPase Activity

**Figure 5:**
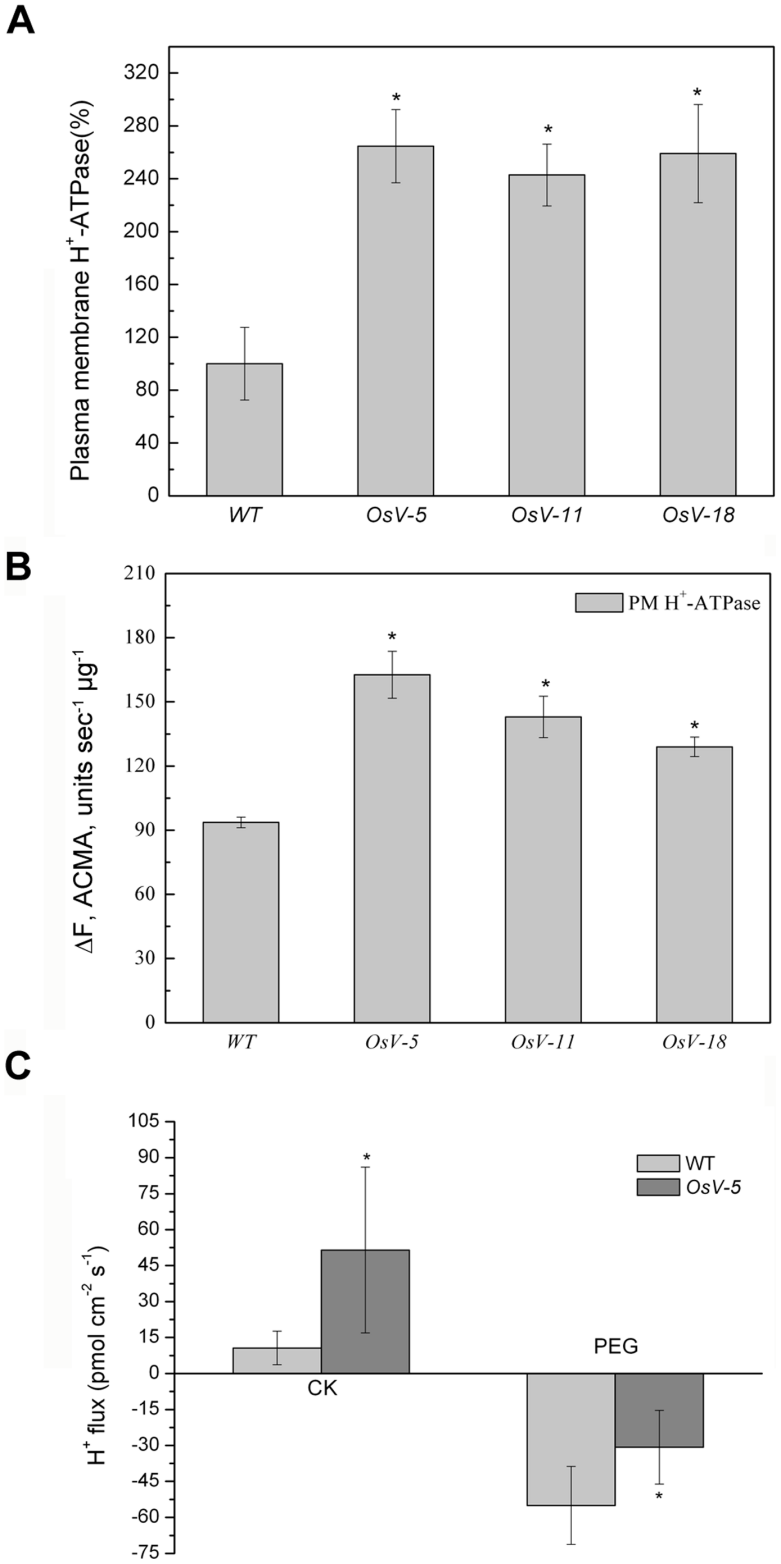
The H^+^ efflux in *OsVHA* *-* *A*-RNAi lines. (A) Plasma membrane H^+^-ATPase activity was determined in wild type (WT) and three *OsVHA-A* RNA interference lines (*OsV-5*, *OsV-11*, and *OsV-18*). (B) Plasma membrane H^+^-ATPase proton-pumping measured by the quenching of ACMA fluorescence. Ten micrograms of tonoplast vesicles were applied to detect fluorescence density. (C) The H^+^ fluxes determined by micro-test system in wild type and transgenic line (*OsV-5*) under normal (CK) and 20% PEG6000 treatment (PEG). The positive values mean ion effluxes, whereas negative values show ion influx. Values are means ± SE (n  = 6). Asterisks (*) indicate significant differences from WT at *P*<0.05.

To evaluate the effect of knockdown of *OsVHA-A* on H^+^ efflux, the activity of plasma membrane H^+^-ATPase was assayed. Plasma membrane H^+^-ATPase in all *OsVHA-A* RNAi transgenic lines had significantly higher activity compared with WT (Figure 5A). In addition, the data of plasma membrane H^+^-ATPase proton-pumping activity indicated that knockdown of *OsVHA-A* enhanced the proton-pumping activity of plasma membrane H^+^-ATPase in contrast with WT (Figure 5B). To further verify the enhanced H^+^ efflux in *OsVHA-A* RNAi repression lines, we examined the extracellular H^+^ concentration by non-invasive micro-test in 1-week-old seedlings (Figure 5C). *OsV-5* was selected as a representative RNAi line for this assay. It was found that the H^+^ efflux in *OsV-5* was 4.8 times more than that of WT in normal condition.

### Knockdown of *OsVHA* ***-*** *A* Results in Increased Stomatal Aperture and Density

**Figure 6:**
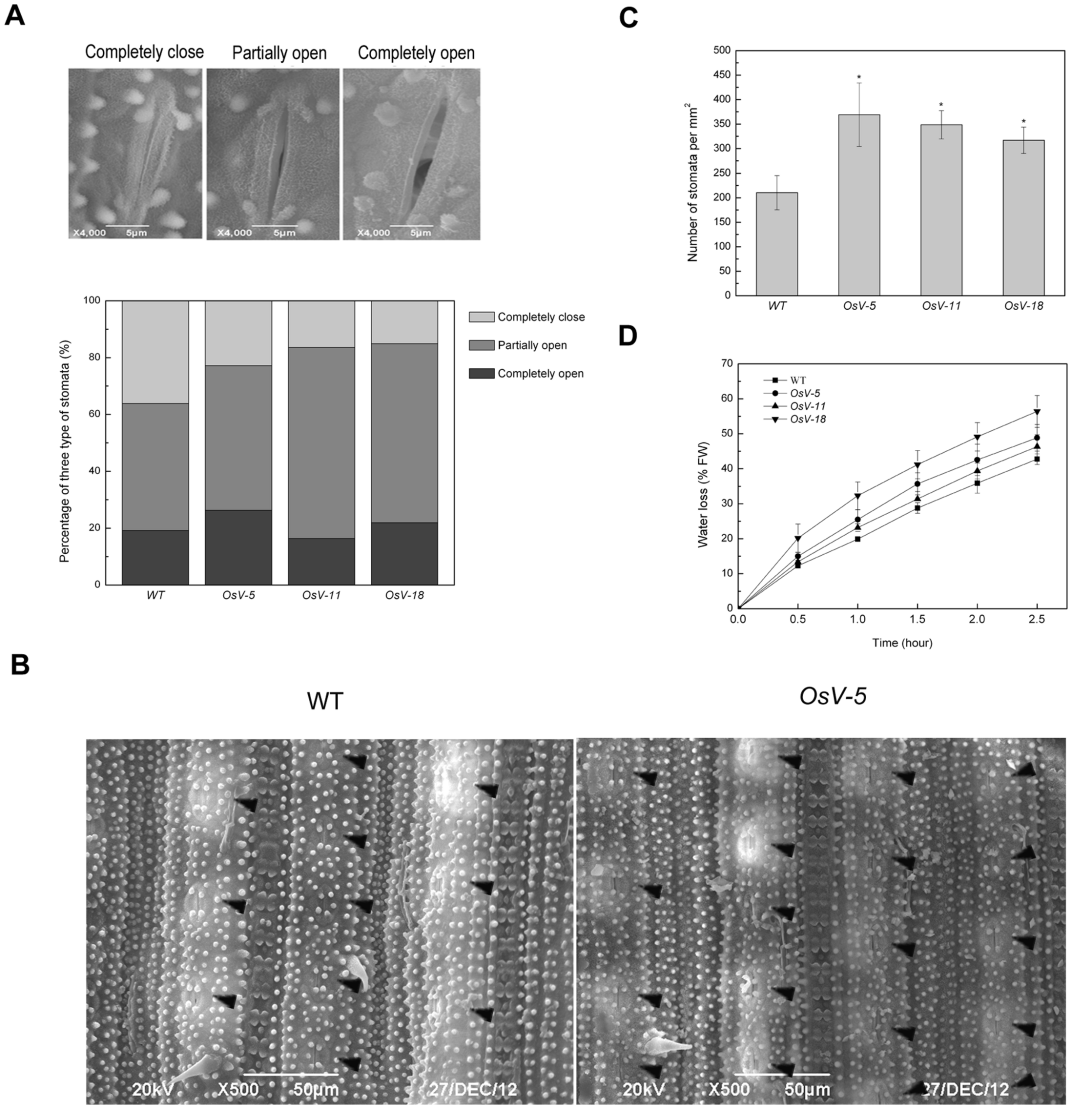
Scanning electron microscopy (SEM) analysis of stomatal apertures and water loss rates in *OsVHA* *-* *A*-RNAi lines. (A) **S**canning electron microscopy images at three different opening levels (completely close, partially open, and completely open) of rice stomata (upper part). Bars  = 5 μM. The stomata percentage at three levels in wild type (WT) and transgenic lines (*OsV-5*, *OsV-11*, and *OsV-18*) were measured (n = 150) (lower part). (B) SEM images (500×) of stomata in the middle of leaves from 3-week-old WT and *OsV-5* (as representative of the transgenic lines) are shown. Stomata are marked by triangle (◂). Bars  = 50 μM. (C) Stomata numbers per mm^2^ in 10 leaves from WT and transgenic plants, respectively, were counted to indicate stoma densities. Asterisks (*) indicate significant differences from WT at *P*<0.05. (D) Comparison of water loss between WT and transgenic plants.

To test whether loss-of-function of *OsVHA-A* alters stomatal development and movement in rice, a comparison of stomatal aperture and density was performed between 3-week-old WT and RNAi seedling leaves by scanning electron microscopy. The observed stomata could be classified as completely open, partially open and completely closed in leaves of both WT and RNAi plants. Compared to 36.17% completely closed and 19.149% completely open stomata in WT leaves, we observed 22.807%, 16.394% and 15.068% completely closed and 26.316%, 16.393% and 21.918% completely open stomata in *OsV-5, OsV-11* and *OsV-18* leaves, respectively (Figure 6A). Intriguingly, 50-75% denser stomata were visualized in 3-week-old *OsVHA-A* RNAi plants than in WT plants (Figure 6B and C).

The enhanced stomatal aperture in the RNAi lines was further examined by water loss analysis. To this end, detached intact leaves from 3-week-old seedling lied on room temperature and the fresh weight was measured every half hour for a 2.5-hour time periods. The analysis of water loss during dehydration stress indicated more water loss in 3-week-old RNAi lines than in WT (Figure 6D). These results suggest that knockdown of *OsVHA-A* leads to remarkably increased stomatal aperture and density.

### RNAi Repression of *OsVHA* ***-*** *A* in Transgenic Rice Enhances Sensitivity to Salt Stress

**Figure 7:**
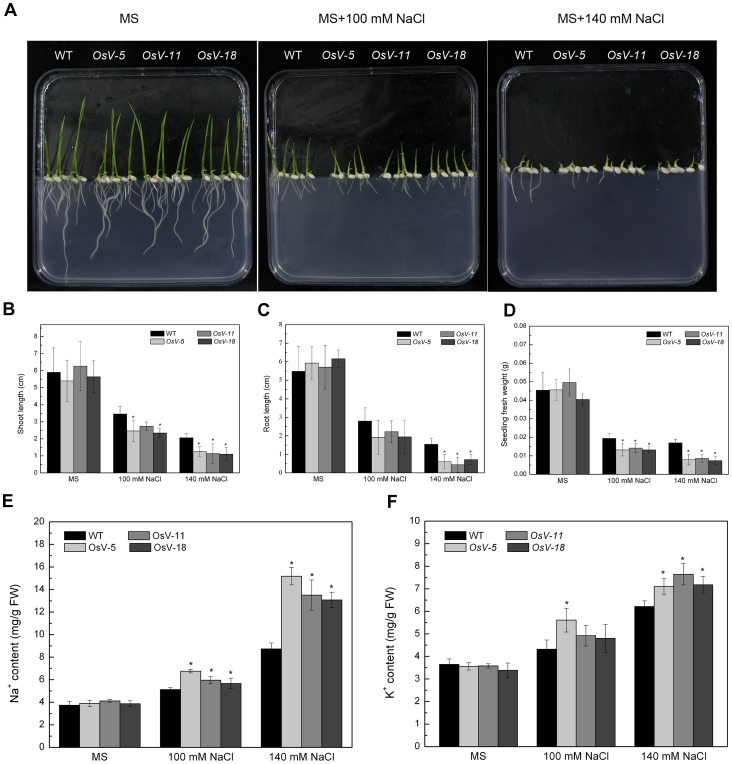
Phenotypes of WT and transgenic seedlings grown on MS media adding different concentrations of NaCl. (A) One-week-old wild type (WT) and transgenic (*OsV-5*, *OsV-11*, *OsV-18*) seedlings grown on MS media with 0, 100, 140 mM NaCl, respectively. Results shown are representative. Shoot length, root length, and fresh weight were shown in (B), (C), and (D), respectively. Contents of Na^+^ (E) and K^+^ (F) in WT and transgenic seedlings from (A) were shown. Asterisks (*) indicate significant differences from WT at *P*<0.05.

To evaluate the effects of knockdown of *OsVHA-A* on the growth of plants under stress conditions, WT and RNAi (*OsV-5*, *OsV-11* and *OsV-18*) seeds were germinated on MS media containing different concentrations of NaCl (0, 100, 140 mM). As shown in Figure 7A, the growth was much severely inhibited in the RNAi seedlings than in the WT seedlings. At 7 DAG on MS medium supplemented with 100 or 140 mM NaCl, the shoot length of WT seedlings reduced to 58.6% and 34.9% of that of the control seedlings grown on normal MS medium; whereas the shoot length of RNAi seedlings reduced to 41.5-45.6% and 18.2-23.3% of those of the control seedlings grown on normal MS medium (Figure 7B). Meanwhile, the root length of WT seedlings was reduced to 51.1% and 28.1% of that of the control seedlings grown on normal MS medium, whereas the root length of RNAi seedlings reduced to 31.5-38.9% and 7.7-11.7% of those of the control seedlings grown on normal MS medium (Figure 7C). The fresh weight of WT seedlings grown on MS medium supplemented with 100 or 140 mM NaCl reduced to 42.7% and 37.4% of control seedlings grown on normal MS medium, whereas the fresh weight of RNAi seedlings reduced to 28.6-32.7% and 17.3-18.3% of those of the control seedlings grown on normal MS medium (Figure 7D). A similar result was also observed in 3-week-old seedlings grown for 12 days in hydroponic culture supplemented with 140 mM NaCl (Figure S4A). The shoot fresh weight of transgenic lines only accounted for 39.9-66.4% of that of the WT seedlings under the 140 mM NaCl condition (Figure S4B). These results suggest that *OsVHA-A* RNAi repression lines shows a significantly increased sensitivity to salt stress.

To decipher the nature of salt stress sensitivity in *OsVHA-A* RNAi transgenic lines, the content of intracellular K^+^ and Na^+^ was determined. One-week-old seedlings grown on MS media containing 0, 100, or 140 mM NaCl were examined. Both K^+^ and Na^+^ contents showed a significant increase in *OsVHA-A* RNAi lines compared to the WT seedlings (Figure 7E and F). In addition, when 3-week-old seedlings were grown on MS medium supplemented with 140 mM NaCl for 12 days, a significant increase in both K^+^ and Na^+^ content was also detected in the shoot of *OsVHA-A* RNAi lines compared with the WT seedlings. These results suggest that the enhanced sensitivity of *OsVHA-A* RNAi lines might be due to an accumulation of Na^+^ toxicity as well as a disruption of osmotic adjustment (Figure S4C and D).

### Knockdown of *OsVHA* ***-*** *A* in Transgenic Rice Increases Susceptibility to Drought Stress

**Figure 8:**
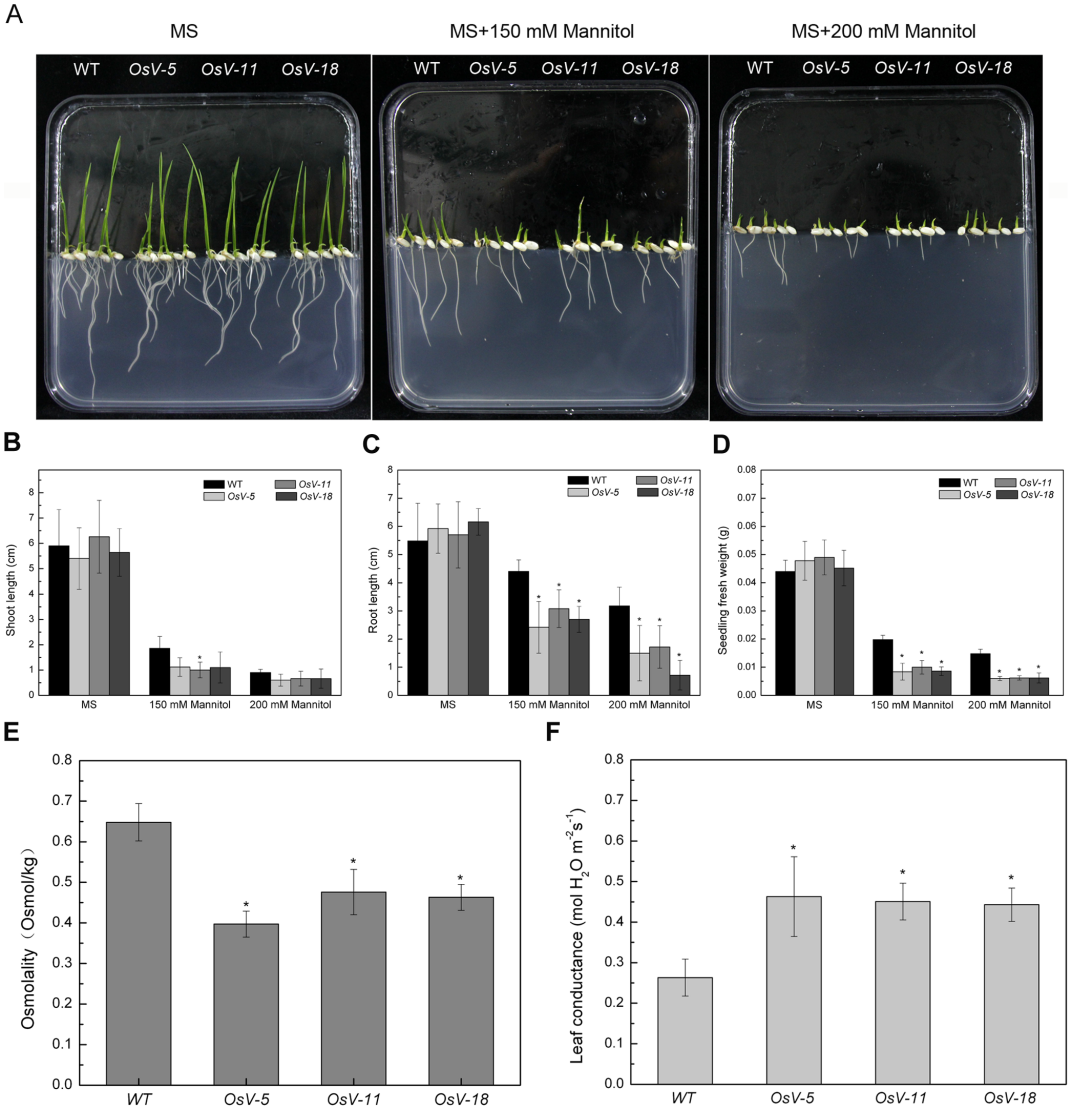
Phenotypes of WT and transgenic seedlings grown on MS media with different concentrations of mannitol. One-week-old wild type (WT) and transgenic (*OsV-5*, *OsV-11*, *OsV-18*) seedlings grown on MS media with 0, 150, 200 mM mannitol, respectively. Results shown are representative. Shoot length, root length, and fresh weight were shown in (B), (C), and (D), respectively. Twenty-day-old wild type (WT) and transgenic plants (*OsV-5*, *OsV-11*, *OsV-18*) were treated with or without 20% PEG6000 for 21 d. Osmolality (C) and leaf conductance (D) from 10 fully expanded leaves of these plants were measured. Asterisks (*) indicate significant differences from WT at *P*<0.05.

To further determine whether downregulation of *OsVHA-A* affects drought tolerance in plants, WT or transgenic seeds were germinated on MS medium containing different concentrations of mannitol (0, 150, 200 mM) (Figure 8A). At 7 DAG on MS medium supplemented with 150 or 200 mM mannitol, the shoot length of WT seedlings reduced to 31.5% and 15.3% of that of the control seedlings grown on normal MS medium, respectively, whereas the shoot length of transgenic seedlings reduced to 16-20.7% and 10.5-11.7% of those of the control seedlings grown on normal MS medium (Figure 8B). Similar effect was observed on roots. The root length of WT seedlings reduced to 80.2% and 58% of the control seedlings grown on normal MS medium, whereas the root length of RNAi seedlings reduced to 40.9-54% and 11.7-30.2% of those of the control seedlings grown on normal MS medium (Figure 8C). In addition, the fresh weight of WT seedlings grown on MS medium supplemented with 150 or 200 mM mannitol reduced to 45.1% and 33.6% of that of the control seedlings grown on normal MS medium, whereas the fresh weight of RNAi seedlings reduced to 17.6-20.4% and 12.6-13.7% of those of the control seedlings grown on normal MS medium (Figure 8D).

Moreover, the altered tolerance of RNAi seedlings was also determined by PEG6000 (see materials and methods for experimental details). We found that, in comparison with WT seedlings, *OsVHA-A* RNAi seedlings displayed more severe wilting (Figure S5A) and had shorter shoot length that only accounted for about 80% of that of WT (Figure S5B). These results suggest that *OsVHA-A* RNAi repression lines have significantly increased sensitivity to osmotic stress.

**Figure 9:**
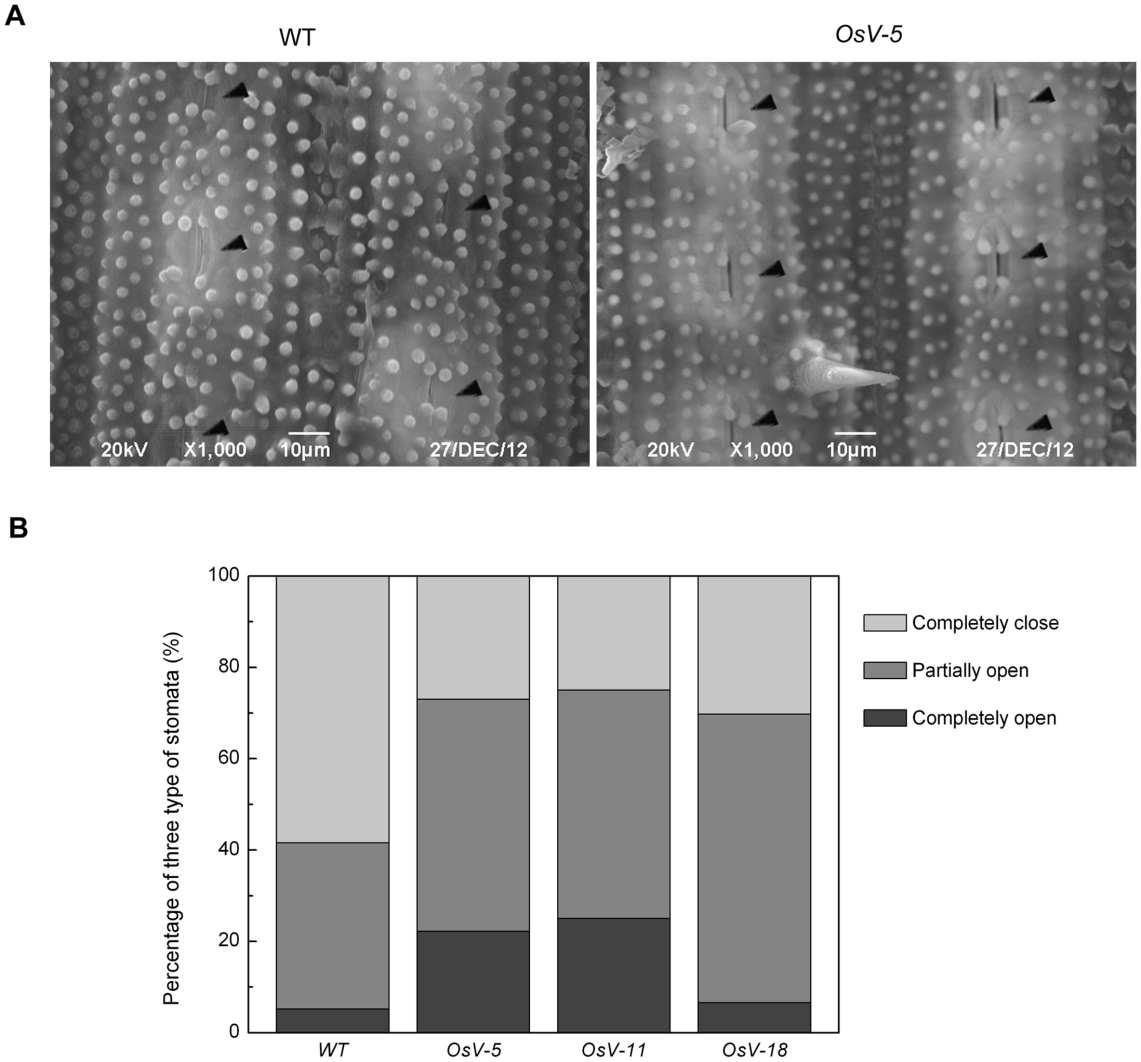
Scanning electron microscopy (SEM) analysis of stomatal apertures in RNAi lines and WT under drought. (A) Leaves of 3-week-old plants treated with 20% PEG6000 for 21 days were used to determine the stomatal aperture. SEM images (1000×) of stomata from WT and *OsV-5* transgenic plants are presented. Stomata are marked by triangle (◂). Bars  = 10 μM. (B) The stomata percentages at three levels in wild type (WT) and transgenic lines (*OsV-5*, *OsV-11*, and *OsV-18*) under 2-week drought stress were measured (n  = 150).

To elucidate the physiological mechanism of drought sensitivity in *OsVHA-A* repression lines, the osmolality and leaf conductance were examined. Leaves were harvested from 3-week-old seedlings grown in hydroponic culture containing 20% PEG6000. The leaf osmolality of RNAi lines reduced to 61.3-73.4% of that of WT under 20% PEG6000 (Figure 8E). The result suggests that the enhanced drought sensitivity in RNAi lines is due to a low solute potential. In addition, the leaf conductance of RNAi lines increased to 168.3-175.8% of that of WT under 20% PEG6000 (Figure 8F). The results indicated that *OsVHA*-*A*-RNAi might affect stomatal movement under osmotic stress. To verify this speculation, the stomatal apertures were determined by scanning electron microscopy with 3-week-old seedling after 2-week drought treatment. 58.442% stomata were completely closed in WT, but only 25-30.3% stomata were completely closed in RNAi leaves (Figure 9). These results indicated that *OsVHA*-*A*-RNAi suppressed the closure of stomata in response to stress conditions.

As shown in Figure 5C, the H^+^ influx of *OsV-5* was 55.85% of that in WT. *OsVHA*-RNAi might repress the H^+^ influx and influence the ion transport, consequently changing the intracellular osmolality and resulting in enhanced stomatal conductance and the sensitivity of osmotic stress.

### Alteration of Downstream Gene Expression Caused by Downregulation of *OsVHA* ***-*** *A*

**Figure 10:**
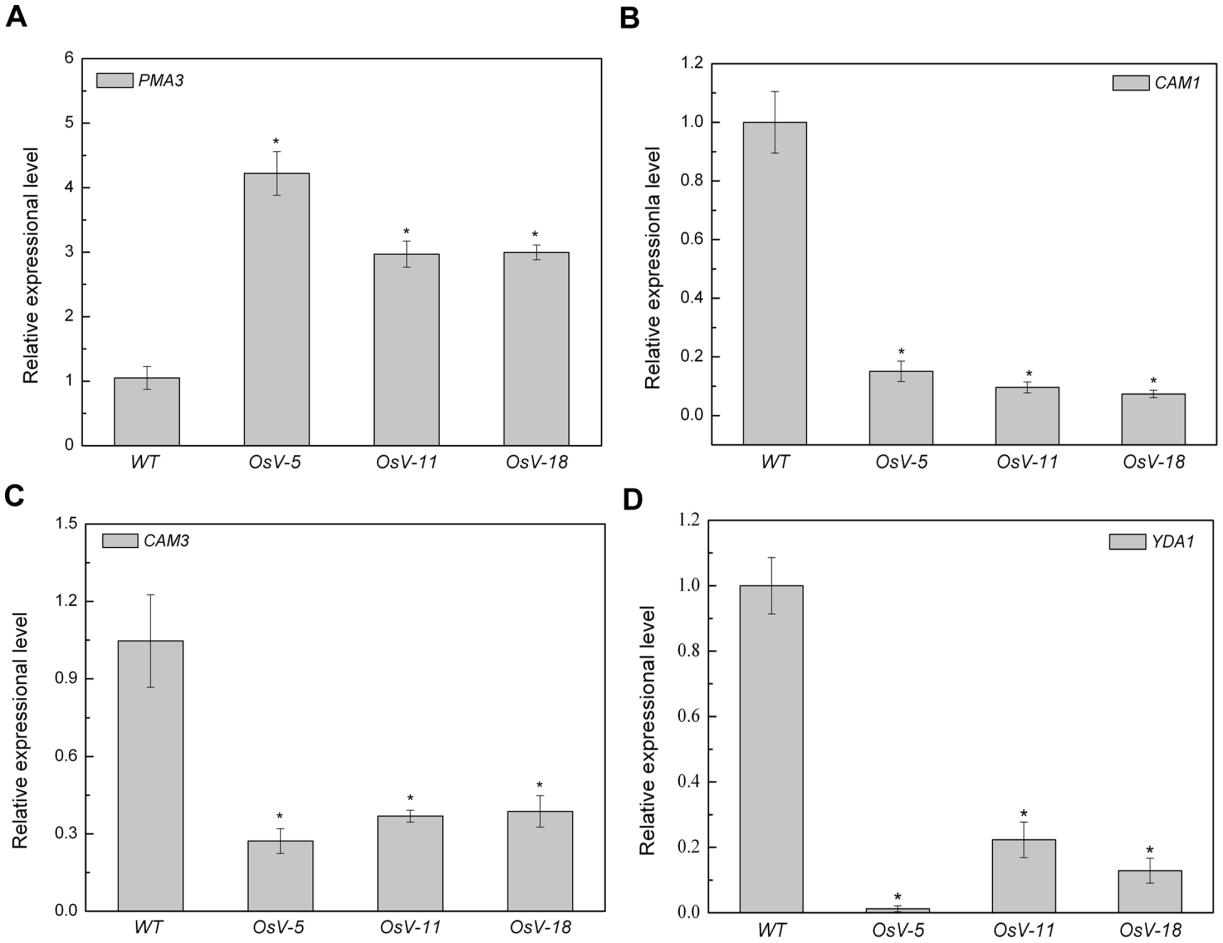
Alteration of gene expression resulting from downregulation of *OsVHA* *-* *A*. Real-time quantitative RT-PCR analysis of mRNA levels of *PMA3* (A), *CAM1* (B), *CAM3* (C), *YDA1* (D) in wild-type (WT) and three independent Os-VHA-A deficient lines (*OsV-5*, *OsV-11*, and *OsV-18*). Asterisks (*) indicate significant differences from WT at *P*<0.05.

Next we examined the effect of downregulation of OsVHA-A on genes involved in the stomatal development and movement. As the alteration of vacuolar pH may result in the adjustment of *PMA* expression, we examined the expression of one member of *PMA* gene family (*PMA3*). As shown in Figure 10A, the expressional level of *PMA3* was significantly up-regulated in 1-week-old *OsVHA*-*A*-RNAi lines. In addition, the alteration of cellular pH value also causes the adjustment of some Ca^2+^-responsive genes expression [60]. Thus, calmodulin as a well-known transducer of Ca^2+^ signals, which was functioned in controlling the stomatal closure, was investigated as well [61]. As shown in Figure 10B and C, *CAM1* and *CAM3*, encoding calmodulin, were significantly down-regulated in 1-week-old *OsVHA*-RNAi lines. Moreover, *YDA1*, encoding a member of MAPKs involved in the regulation of stomatal development [9], was also significantly down-regulated in all three *OsVHA*-*A*-RNAi lines (Figure 10D), suggesting that *OsVHA* might control the stomatal density via regulating *YDA1* expression.

## Discussion

Many genes have been shown to be involved in the regulation of stomatal movement [3]. However, it is unclear whether V-ATPase subunit A has a role in regulating this process. In this study, a homologous gene encoding the V-ATPase subunit A was identified in rice. By using RNAi technique, we generated three transgenic plants expressing 35S-*OsVHA-A*-RNAi construct. A comparison analysis of stomatal aperture between WT and the RNAi transgenic plants revealed that knowdown of *OsVHA-A* promotes the expand of stomatal aperture. In addition, a denser population of stomata was observed in the RNAi transgenic plants (Figures 6 and S6A). It appears that the *OsVHA-A* regulates stomatal density and aperture via interfering with pH value and ionic equilibrium in guard cells thereby affecting the growth of rice plants under normal condition as well as salt and osmotic stress conditions.

To systemically study the mechanism of *OsVHA-A* functioned in regulation of stomatal movement, subcellular localization was examined by microscopy and we found that OsVHA-A was exclusively localized on tonoplast even under overexpression condition (Figure 1B). Although the tomato VHA-A was found to be localized in Golgi too, a functional proton-pumping was only observed on tonoplast in tomato [62]. Together, these results indicate a possible role of OsVHA-A in regulation of vacuolar function. Supporting this notion, heterologous expression of *OsVHA-A* nearly rescued the yeast mutant (*vma1Δ*) phenotype (lacking of V-ATPase subunit A activity) in high pH value medium (Figure 2), and knockdown of *OsVHA-A* in rice transgenic plants significantly inhibited the V-ATPase activity (Figure 4). Similar result was also found in Arabidopsis mutant of subunit VHA-a [31]. V-PPase, another proton pump, is also localized on tonoplast, which was thought to be a backup system for V-ATPase in the case of ATP deficiency [20]. However, knockdown of OsVHA-A did not affect the activity of V-PPase. A similar result is found in the AtVHA-a3 mutant, in which lack of *AtVHA-a* down-regulates the activity of V-ATPase but not that of V-PPase [31], suggesting that OsVHA-A specifically regulates the activity of V-ATPase. It is possible that the effect of knockdown of *OsVHA-A* on the stomatal development and movement is through the alteration of V-ATPase activity.

V-ATPase has been demonstrated to function in vacuolar acidification and pH gradient establishment across the vacuolar membrane [63]. We also observed a significant increase of the vacuolar pH and reduction of proton-pumping activity in *OsVHA-A* RNAi transgenic rice plants compared to the WT rice plants. To maintain the intracellular H^+^ homeostasis, the disturbance of H^+^ transport from cytosol to vacuole might result in acceleration of H^+^ efflux from cytoplasm to extracellular region, which is confirmed by the finding that an efflux of H^+^ is more in *OsVHA-A* RNAi plants than that of WT plants under normal condition and the proton-pumping activity of plasma membrane H^+^-ATPase was up-regulated in *OsVHA-A* repression lines compared with WT (Figure 5B and C). An increased *PMA3* expression and plasma membrane H^+^-ATPase activity resulting from downregulation of *OsVHA-A* might account for the acceleration of H^+^ efflux in the transgenic plants (Figures 10A and 5A). Additionally, we also found the content of intracellular K^+^ in *OsVHA-A* RNAi plants is more than that in WT plant under salt stress (Figures 7F and S5D), which is consistent with previous observation that the efflux of H^+^ promotes the hyperpolarization of plasma membrane [24], resulting in acceleration of K^+^ influx. In addition, K^+^ uptake plays a major role in modulating guard cell turgor and volume [3], thereby regulating enlargement of stomatal conductance and expand of stomatal aperture. Consistent with this speculation, an elevated stomatal conduction and enlarged aperture were found in transgenic plants compared to the WT plants under drought stress (Figures 8E & F, 9 and S6B). Thus, we speculate that knockdown of *OsVHA-A* triggers the H^+^ efflux and the hyperpolarization of plasma membrane, which enhances the uptake of K^+^, resulting in the expand of stomatal aperture.

Actually, the effect of the pH value on stomatal movement has been reported previously. In the plant cell, intracellular H^+^ homeostasis is dependent on three different proton pumps, PM H^+^-ATPase, V-PPase and V-ATPase [18]. Each of them appears to involve in guard cell signal transduction but display different effects on stomatal movement. Activated PM H^+^-ATPase promotes H^+^ efflux as a result of hyperpolarization of plasma membrane and combined with K^+^ influx and stomatal opening [24]. Nevertheless, V-PPase as well as V-ATPase seem to induce stomatal closures. Overexpression of *AVP1* (*Arabidopsis vacuolar H^+^-PPase*) induces stomatal closure and reduces the number of vacuoles in guard cells [64]. In addition, the subunits C and c1 of V-ATPase have been shown to regulate stomatal movement [33], [34]. The PM H^+^-ATPase mainly provides driving force for the K^+^ influx from extracellular region to cytosol while V-PPase and V-ATPase support the K^+^ efflux from vacuole to cytosol. However, it appears connection exists among these three proton pumps. Our data suggest that knockdown of *OsVHA-A* reduces the activity of V-ATPase, but increase the activity of PM H^+^-ATPase and has no significant effect on the activity of V-PPase, which contributes to the expand of stomatal aperture.

In addition, a significantly increased stomatal density was found in *OsVHA*-*A* RNAi repression plants. Interestingly, accompanied with the knockdown of *OsVHA*-*A*, the expression of a *YDA1* homolog in rice appeared to be repressed (Figure 10D). YDA, as a member of MAPK gene family, has been reported as a repressor in control of cell division and cell fate specification during stomatal development [9]. We could propose that repression of *OsVHA-A* enhanced the stomatal density partially through repressing *YDA1* gene expression. Consistent with these observations, overexpression of a halophyte grass (*spartina alterniflora*) gene *VHA-c1* in rice plants resulted in a significant decrease in stomatal density [34]. Nevertheless, the precise mechanism remains to be investigated.

**Figure 11:**
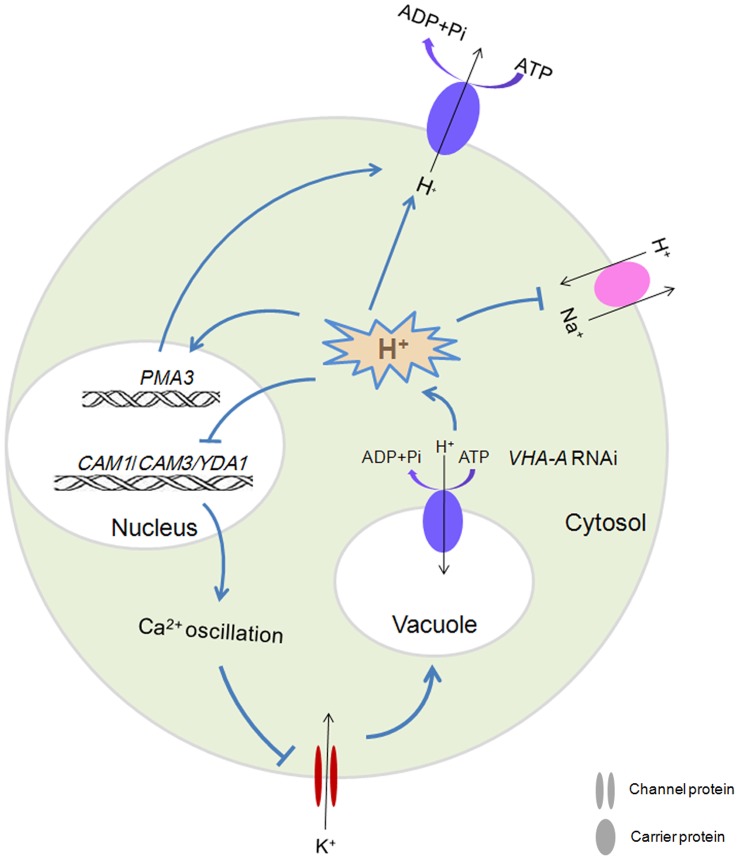
Schematical model of *OsVHA-A*-RNAi in the regulation of stomatal aperture.

A possible signaling pathway to decipher the nature of *OsVHA-A* RNAi-induced increase of stomatal conductance was presented in Figure 11. Repression of *OsVHA-A* expression may lead to a decrease of V-ATPase activity. The decrease of this enzyme activity promoted *PMA3* gene expression and activated the pump of plasma membrane H^+^-ATPase, resulting in the acceleration of H^+^ efflux and K^+^ influx. Moreover, the decrease of V-ATPase activity resulted in downregulation of *CAM1*, *CAM3* and *YDA1* that have been demonstrated to be involved in regulation of stomatal opening and density. Taken together, *OsVHA-A* may control the stomatal conductance via regulating ionic equilibrium of proton pump and downstream gene expression.

## Supporting Information

Figure S1 Amino acid alignment among OsVHA-A homologues. Multiple amino acid sequences alignment of OsVHA-A homologues derived from *Homo sapiens* (accession no. NM_001690.3), *Mus musculus* (accession no. NM_007508.5), *Danio rerio* (accession no. XM_002666640.2), *Saccharomyces cerevisiae* (accession no. AF389404.1), *Arabidopsis thaliana* (accession no. NM_001036222.2), *Glycine max* (accession no. NM_001255132.2), *Sorghum bicolor* (accession no. XM_002451594.1), *Triticum aestivum* (accession no. AK332978.1), *Vitis vinifera* (accession no. XM_002267243.2), *Zea mays* (accession no. AY104754.1), *Oryza sativa* (accession no. NM_001064815.1). The identical residues are shaded by black in white letters. Residues with at least 75% identity are shaded by deep gray in black letters. Residues with at least 50% identity are shaded by light gray in black letters. Amino acid residue numbers are indicated on the right.
(TIF)

Figure S2 Phylogenetic analysis of OsVHA-A homologues.
(TIF)

Figure S3 Calibration of vacuolar pH measurement. *In situ* calibration was used to determine the vacuolar pH values. The fluorescence ratios (488/458 nm) were plotted against the pH of the equilibration buffers to obtain a calibration curve. Error bars show SE of the mean with n  = 15 seedlings.
(TIF)

Figure S4 The transgenic plants exhibiting decreased tolerance to the stress of 140 mM NaCl. (A) Twenty-day-old wild type (WT) and transgenic (*OsV-5*, *OsV-11*, *OsV-18*) plants were treated with or without 140 mM NaCl for 12 d. Results shown are representative. Shoot fresh weights of (A) were shown in (B). Na^+^ and K^+^ contents (C and D, respectively) were measured. Asterisks (*) indicate significant differences from WT at *P*<0.05.
(TIF)

Figure S5 The transgenic plants showing elevated sensitivity under 20% PEG6000 treatment. (A) Twenty-day-old wild type (WT) and transgenic (*OsV-5*, *OsV-11*, *OsV-18*) plants were treated with or without 20% PEG6000 for 21 d. Results shown are representative. Shoot length (B) from 10 fully expanded leaves of plants in (A) were shown. Values are means ± SE (n  = 6). Asterisks (*) indicate significant differences from WT at *P*<0.05.
(TIF)

Figure S6 Scanning electron microscopy (SEM) analysis of stomatal apertures in RNAi lines and WT under normal and drought conditions. (A) SEM images (500×) of stomata in the middle of leaves from 3-week-old WT and transgenic plants are shown. Stomata are marked by triangle (◂). Results of *OsV-11 and OsV-18* shown are representative. Bars  = 50 μM. (B) Leaves of 3-week-old plants treated with 20% PEG6000 for 21 days were used to determine the stomatal aperture. SEM images (1000×) of stomata from WT, *OsV-11* and *OsV-18* transgenic plants are shown. Stomata are marked by triangle (◂). Bars  = 10 μM.
(TIF)
