# Supplementary figures and images for: RNAi-Directed Downregulation of Vacuolar H+ -ATPase Subunit A Results in Enhanced Stomatal Aperture and Density in Rice
Source: PLoS One. 2013 Jul 22;8(7):e69046. doi: 10.1371/journal.pone.0069046 (PMC3718813; doi:10.1371/journal.pone.0069046)

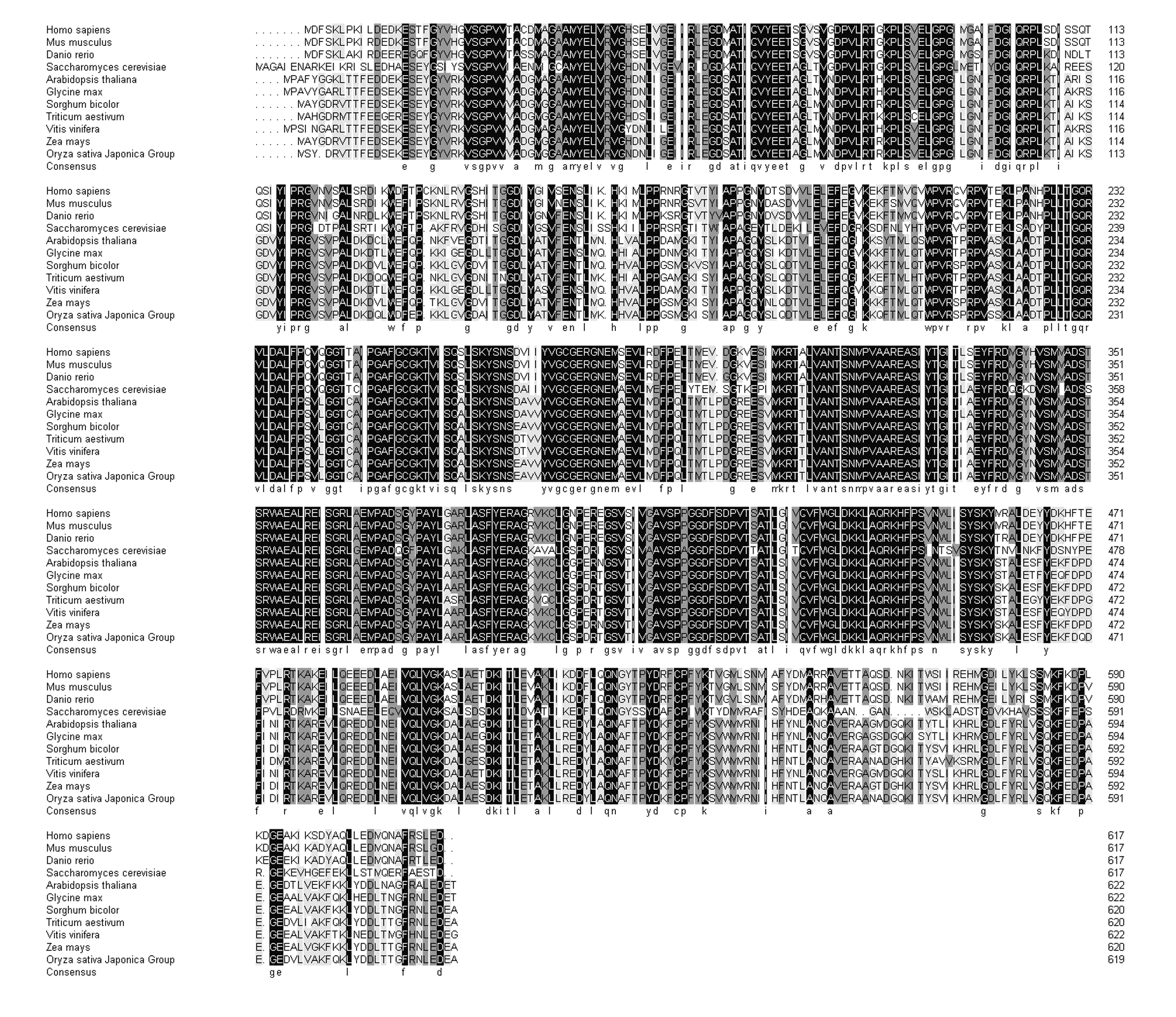

Supplement: Figure S1 — Amino acid alignment among OsVHA-A homologues. Multiple amino acid sequences alignment of OsVHA-A homologues derived from Homo sapiens (accession no. NM_001690.3), Mus musculus (accession no. NM_007508.5), Danio rerio (accession no. XM_002666640.2), Saccharomyces cerevisiae (accession no. AF389404.1), Arabidopsis thaliana (accession no. NM_001036222.2), Glycine max (accession no. NM_001255132.2), Sorghum bicolor (accession no. XM_002451594.1), Triticum aestivum (accession no. AK332978.1), Vitis vinifera (accession no. XM_002267243.2), Zea mays (accession no. AY104754.1), Oryza sativa (accession no. NM_001064815.1). The identical residues are shaded by black in white letters. Residues with at least 75% identity are shaded by deep gray in black letters. Residues with at least 50% identity are shaded by light gray in black letters. Amino acid residue numbers are indicated on the right. (TIF) [file pone.0069046.s001.tif]

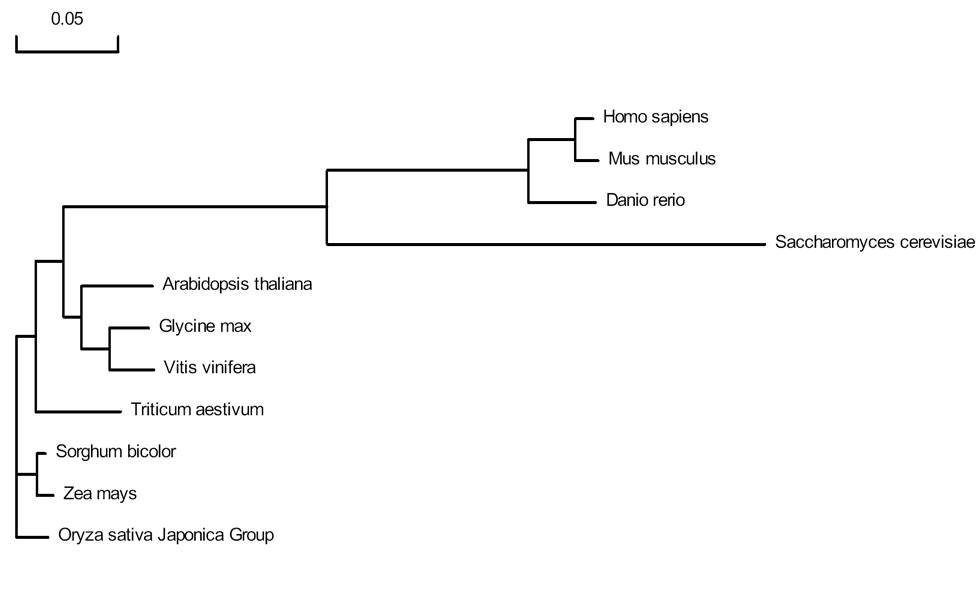

Supplement: Figure S2 — Phylogenetic analysis of OsVHA-A homologues. (TIF) [file pone.0069046.s002.tif]

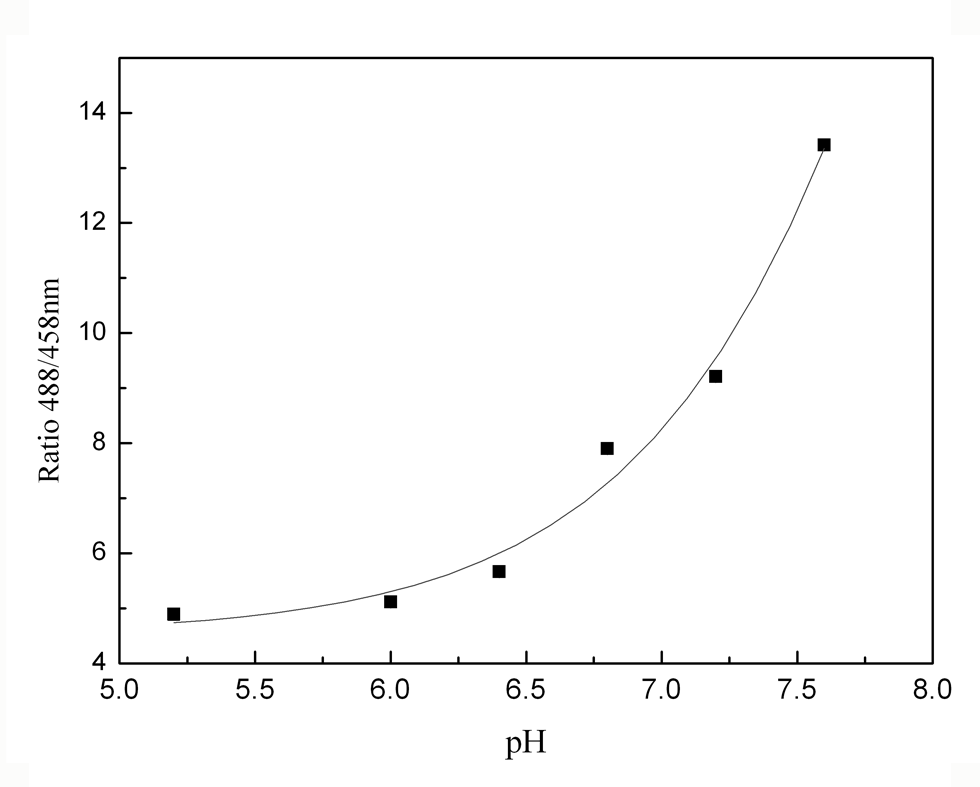

Supplement: Figure S3 — Calibration of vacuolar pH measurement. In situ calibration was used to determine the vacuolar pH values. The fluorescence ratios (488/458 nm) were plotted against the pH of the equilibration buffers to obtain a calibration curve. Error bars show SE of the mean with n = 15 seedlings. (TIF) [file pone.0069046.s003.tif]

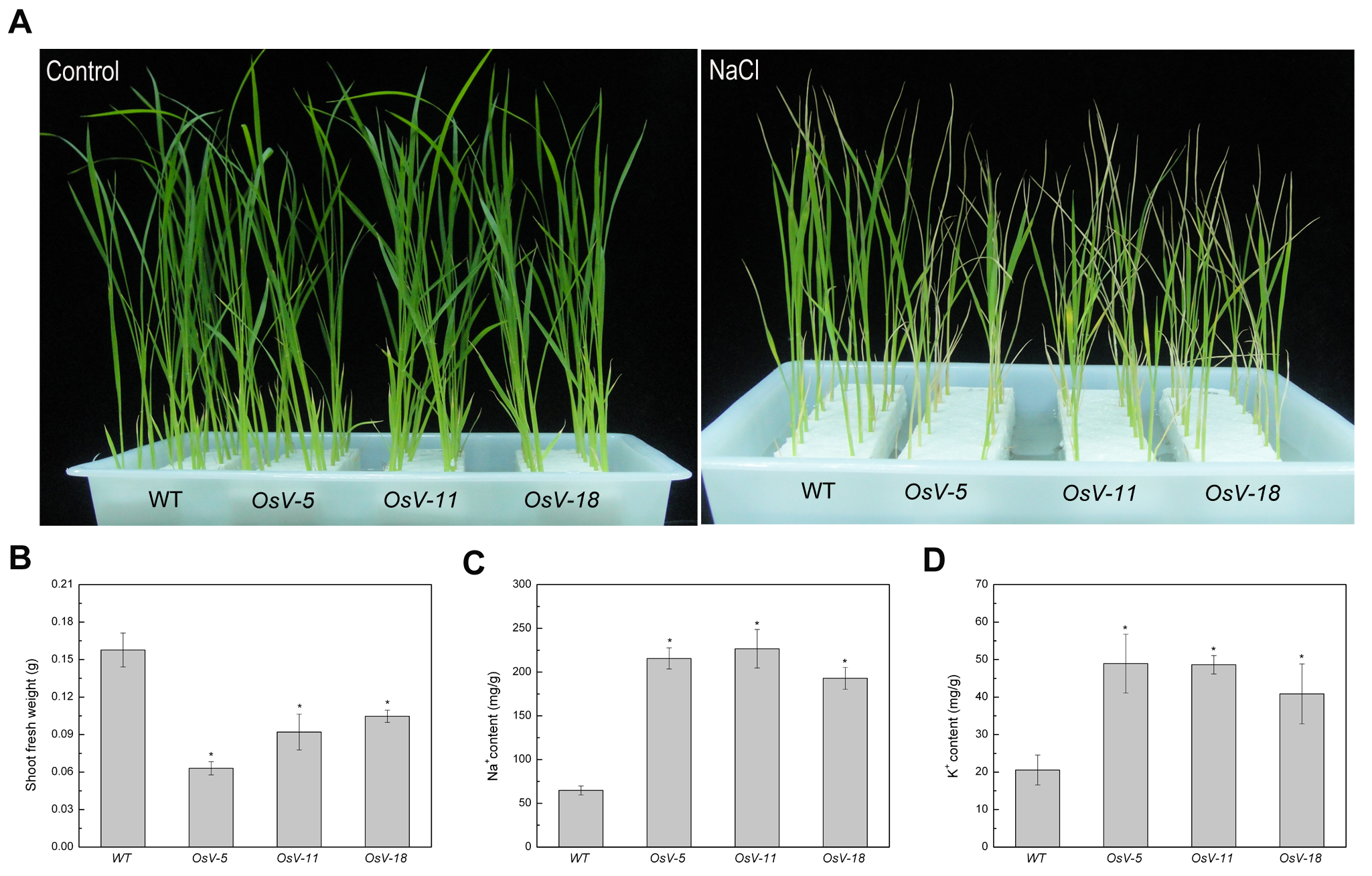

Supplement: Figure S4 — The transgenic plants exhibiting decreased tolerance to the stress of 140 mM NaCl. (A) Twenty-day-old wild type (WT) and transgenic (OsV-5, OsV-11, OsV-18) plants were treated with or without 140 mM NaCl for 12 d. Results shown are representative. Shoot fresh weights of (A) were shown in (B). Na+ and K+ contents (C and D, respectively) were measured. Asterisks (*) indicate significant differences from WT at P<0.05. (TIF) [file pone.0069046.s004.tif]

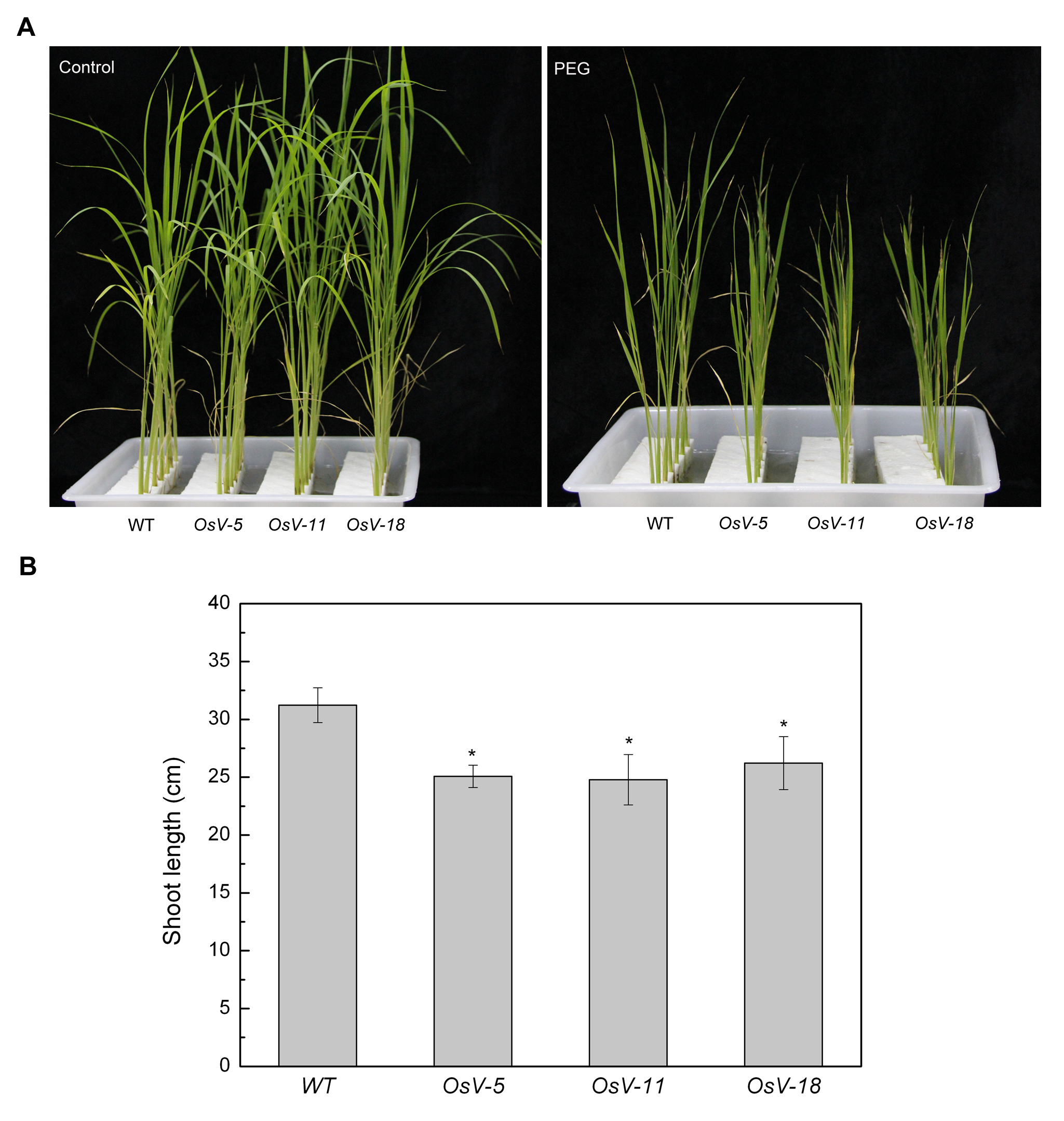

Supplement: Figure S5 — The transgenic plants showing elevated sensitivity under 20% PEG6000 treatment. (A) Twenty-day-old wild type (WT) and transgenic (OsV-5, OsV-11, OsV-18) plants were treated with or without 20% PEG6000 for 21 d. Results shown are representative. Shoot length (B) from 10 fully expanded leaves of plants in (A) were shown. Values are means ± SE (n = 6). Asterisks (*) indicate significant differences from WT at P<0.05. (TIF) [file pone.0069046.s005.tif]

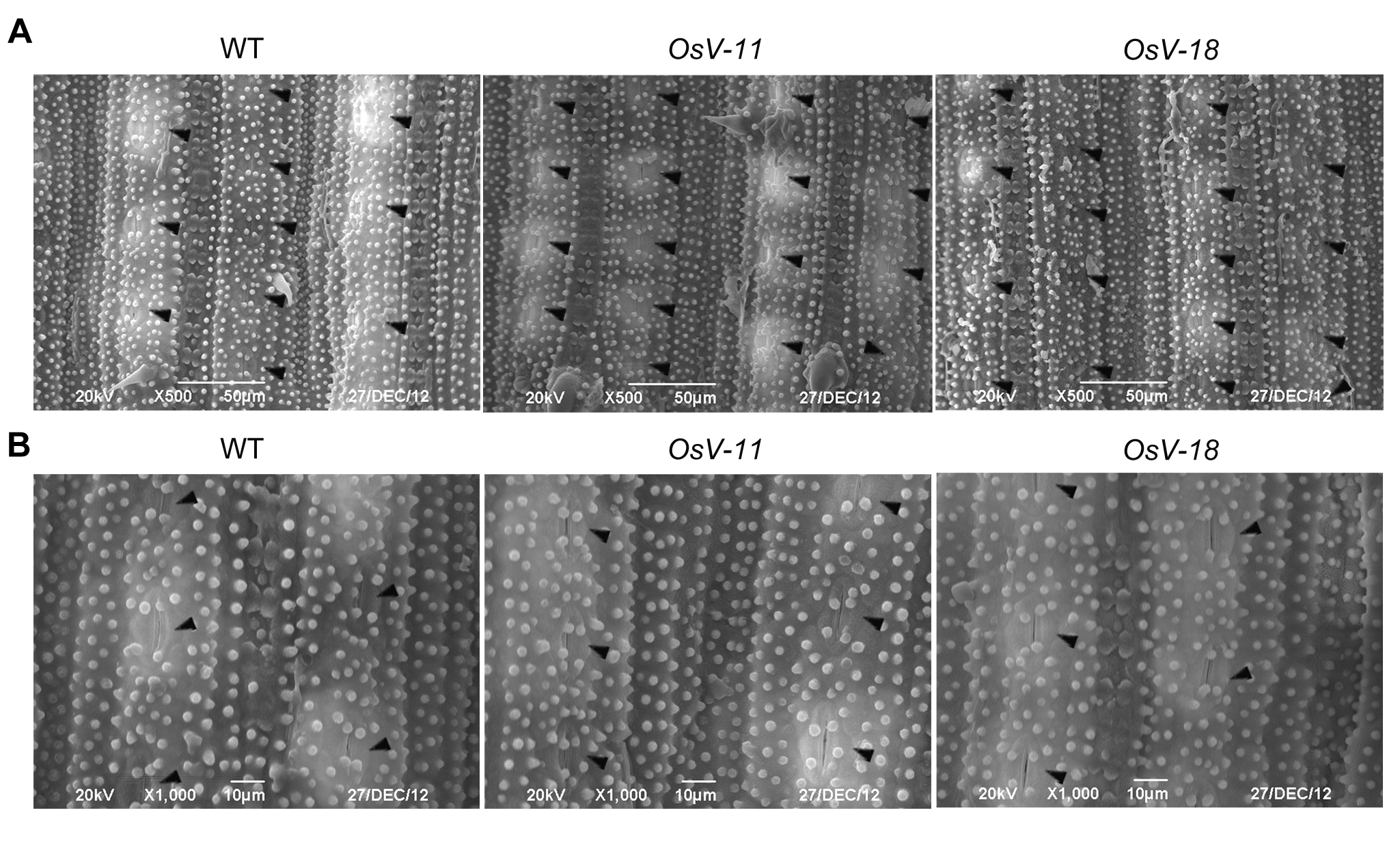

Supplement: Figure S6 — Scanning electron microscopy (SEM) analysis of stomatal apertures in RNAi lines and WT under normal and drought conditions. (A) SEM images (500×) of stomata in the middle of leaves from 3-week-old WT and transgenic plants are shown. Stomata are marked by triangle (◂). Results of OsV-11 and OsV-18 shown are representative. Bars = 50 μM. (B) Leaves of 3-week-old plants treated with 20% PEG6000 for 21 days were used to determine the stomatal aperture. SEM images (1000×) of stomata from WT, OsV-11 and OsV-18 transgenic plants are shown. Stomata are marked by triangle (◂). Bars = 10 μM. (TIF) [file pone.0069046.s006.tif]
